# A Novel Immune-Gene Pair Signature Revealing the Tumor Microenvironment Features and Immunotherapy Prognosis of Muscle-Invasive Bladder Cancer

**DOI:** 10.3389/fgene.2021.764184

**Published:** 2021-11-26

**Authors:** Xiaonan Zheng, Xianghong Zhou, Hang Xu, Di Jin, Lu Yang, Bairong Shen, Shi Qiu, Jianzhong Ai, Qiang Wei

**Affiliations:** ^1^ Department of Urology, West China Hospital, Sichuan University, Chengdu, China; ^2^ Institute of Systems Genetics, West China Hospital, Sichuan University, Chengdu, China; ^3^ Center of Biomedical Big Data, West China Hospital, Sichuan University, Chengdu, China

**Keywords:** muscle-invasive bladder cancer, immune-related gene pair signature, tumor microenvironment, immunotherapy, prognosis

## Abstract

Immunotherapy has been a milestone for muscle-invasive bladder cancer (MIBC), but only a small portion of patients can benefit from it. Therefore, it is crucial to develop a robust individualized immune-related signature of MIBC to identify patients potentially benefiting from immunotherapy. The current study identified patients from the Cancer Genome Atlas (TCGA) and immune genes from the ImmPort database, and used improved data analytical methods to build up a 45 immune-related gene pair signature, which could classify patients into high-risk and low-risk groups. The signature was then independently validated by a Gene Expression Omnibus (GEO) dataset and IMvigor210 data. The subsequent analysis confirmed the worse survival outcomes of the high-risk group in both training (*p* < 0.001) and validation cohorts (*p* = 0.018). A signature-based risk score was proven to be an independent risk factor of overall survival (*p* < 0.001) and could predict superior clinical net benefit compared to other clinical factors. The CIBERSORT algorithm revealed the low-risk group had increased CD8^+^ T cells plus memory-activated CD4^+^ T-cell infiltration. The low-risk group also had higher expression of PDCD1 (PD-1), CD40, and CD27, and lower expression of CD276 (B7-H3) and PDCD1LG2 (PD-L2). Importantly, IMvigor210 data indicated that the low-risk group had higher percentage of “inflamed” phenotype plus less “desert” phenotype, and the survival outcomes were significantly better for low-risk patients after immunotherapy (*p* = 0.014). In conclusion, we proposed a novel and promising prognostic immune-related gene pair (IRGP) signature of MIBC, which could provide us a panoramic view of the tumor immune microenvironment of MIBC and independently identify MIBC patients who might benefit from immunotherapy.

## Introduction

With over 43,000 patients diagnosed every year worldwide, bladder cancer (BC) accounts for 170,000 deaths annually, causing a heavy public health and socioeconomic burden (Patel et al., 2020). As the advanced stage of BC, muscle-invasive bladder cancer (MIBC) makes up to 20–30% of BC and is even more lethal (Chamie et al., 2013). Radical cystectomy and pre- or postoperative adjuvant chemotherapy are usually given to MIBC patients, but the survivorship and cancer-specific mortality are still poorer than in patients with TaT1 and carcinoma *in situ* (Burger et al., 2013). Therefore, different biomarkers have been studied in molecular classification to predict whether disease progression and prognosis of MIBC have been previously reported. For instance, epidermal growth factor receptor 3 (EGFR3) was reported to be overexpressed in basal MIBC and was chemosensitivity-related (Choi et al., 2014a; Choi et al., 2014b). Another research revealed that ERCC2 mutations were mostly enriched in primary vs secondary MIBC and resulted in increased cisplatin sensitivity (Pietzak et al., 2019). A recent consensus summarized different molecular classification systems of MIBC and defined it into six molecular subtypes (Kamoun et al., 2020). However, as the EAU guideline concluded, molecular classification and biomarkers of MIBC are still evolving and have not been validated in routine clinical practice yet (EAU Guidelines, 2020).

In the era of immunotherapy, tumor microenvironment has been a research hotspot for years, and immune cells were reported to be a critical component of it. In fact, immune cells have longed to play a critical role in tumor development and progression (Miao et al., 2019; Walsh et al., 2019; Mohsenzadegan et al., 2020). Therefore, a robust individualized immune signature predicting prognosis is necessary to identify patients who might benefit from immunotherapy. Although several studies have tried to construct prognostic signatures of MIBC, those signatures were not particularly designed to be immune-related gene. Moreover, the limitations of traditional approaches processing RNA-seq or microarray data such as biological heterogeneity and technical biases could not be ignored in those signatures. Hence, the current study aims to utilize an improved technique established by Li et al. (Li et al., 2017) to construct a robust individualized immune-related gene pair (IRGP) signature that can predict the prognosis of MIBC.

## Methods

### Data Preparation

A level three RNA-seq expression data along with clinical information of TCGA-BLCA project were downloaded from the Cancer Genome Atlas (TCGA) in the FPKM workflow type (https://portal.gdc.cancer.gov/, accessed on May 2020). Clinical information of bladder cancer samples was downloaded from UCSC Xena for double-checking. The additional microarray dataset and clinical information were downloaded from another independent dataset GSE31684 in Gene Expression Omnibus (https://www.ncbi.nlm.nih.gov/geo/, GEO) for necessary external validation. The gene expression data of MIBC patients from GSE31684 were normalized using the robust multi-array average (RMA) method from the “affy” package. Patients diagnosed with muscle-invasive bladder cancer (T stage T2–T4) were included for further analysis, and patients without survival reports or survival less than 30 days were excluded to reduce the potential impact from lethal complication.

### Construction of Immune-Related Gene Pair Signature

Immune-related genes (IRGs) were identified from the ImmPort database (Bhattacharya et al., 2014) on May 24th^,^ 2020 (https://immport.niaid.nih.gov). To construct the IRGP signature, we used a pairwise comparison, as described in a previously published literature (Li et al., 2017), among the IRG expression value to assign a score for each IRGP. Specifically, the score was 1 for certain IRGP when the first IRG of this IRGP was out-expressed than the second IRG, and the score was 0 when the expression value of the second IRG was higher. IRGP would be discarded if the score of this IRGP was identical across over 80% samples. Then we performed Cox regression to select prognostic IRGPs as candidates for the subsequent risk model construction. Lasso Cox proportional hazard regression (iteration = 1,000) with 10-fold cross validation (R package “glmnet”) was applied and screened IRGPs to construct the eventual prognostic model of IRGP.

### Prognostic Value of Immune-Related Gene Pair Signature and External Validation

Patients from the training cohort (TCGA-MIBC) were divided into either high or low IGRP risk group based on the risk score they were assigned in the IRGP signature. The optimal cutoff value discriminating the high- and low-risk score was determined by the “Maxstat” algorithm (Ogłuszka et al., 2019), and the accuracy of our model was assessed by the receiver operating curve (ROC) (Martínez-Camblor and Pardo-Fernández, 2018). 1-year, 3-year, and 5-year ROC were presented. The Kaplan–Meier curve was then used to compare the survival between the high- and low-risk score groups. Patients from GSE31684 and IMvigor210 trial (advanced/metastatic bladder cancer treated with immune-checkpoint inhibitor) were also grouped accordingly, and the survival analysis was performed to validate the IGRP signature. Moreover, univariate and multivariate Cox regression were performed to further validate the prognostic value of the IGRP signature along with other clinical parameters such as age, gender, T stage, N stage, and papillary histology. The decision curve analysis (DCA) was also conducted to compare the clinical benefits between the signature-based risk score and other clinical factors.

### Tumor Microenvironment and Function Analysis of IRGP Signature

CIBERSORT is a machine learning method that can quantify the relative abundance of 22 types of infiltrating immune cells in bulk tumor gene expression profiles (Newman et al., 2015). By uploading the normalized gene expression data to the CIBERSORT website (http://cibersort.stanford.edu/) with the default signature matrix at 1,000 permutations, we predicted and compared different immune cell infiltration in the high-risk and low-risk groups. Moreover, the expression of immune-checkpoint genes was compared between groups, and genes with significant difference were displayed. Importantly, data from IMvigor210 were used to calculate the percentage of three immune phenotypes (inflamed, excluded, and desert) (Chen and Mellman, 2017) between groups, and the risk score of patients with different immune response was assessed.

The gene set enrichment analysis (GSEA) of Gene Ontology (GO) and Kyoto Encyclopedia of Genes and Genomes (KEGG) pathways were performed to assess the biological processes that the IRGP signature was involved with. We downloaded the gmt file from GSEA (https://www.gsea-msigdb.org/) and used a R package named “fgsea” to conduct the enrichment analysis, which identified pathways with a minimal number of 15 genes and a maximal number of 500 genes, and repeated 10,000 times. Enrichment outcomes with *p* value less than 0.05 were identified as statistically significant.

## Result

### Construction of IRGP Signature

A total of 430 files were downloaded from the TCGA database including 411 transitional cell papilloma and carcinoma files and 19 normal tissue files. Data from UCSC Xena had clinical information of 454 patients. After data matching and screening, 385 MIBC patients with sufficient clinical information were included. A total of 2,498 IRGs were then obtained from the ImmPort database, which were involved in a variety of immune functions including but not limited to antigen processing and presentation, antimicrobials, cytokine, chemokine, natural killer cell cytotoxicity, BCR signaling, and TCR signaling pathway (Supplementary Table S1). 21,074 IRGPs were matched after pairwise comparison, of which 343 IRGPs were found to be correlated with the prognosis of MIBC (Supplementary Table S2). Lasso Cox regression (R package “glmnet,” iteration = 1,000) eventually selected 45 prognostic IRGPs to build up the IRGP signature. Immune processes and Lasso coefficients of those 45 IRGPs are presented in Table 1.

**TABLE 1:** Immune-related gene pairs in signature construction.

IRG 1	Immune process	IRG2	Immune process	Coefficient	HR	95% CI	*p* Value
CTSE	Antigen processing and presentation	PTN	Cytokines	−0.19344	0.44	0.30–0.66	0.0001
CTSE	Antigen processing and presentation	TNFRSF14	Cytokine receptors	−0.15899	0.40	0.25–0.62	0.0001
MR1	Antigen processing and presentation	PTGER4	Cytokine receptors	0.255,648	1.78	1.28–2.48	0.0007
ICAM1	Antigen processing and presentation	IL20RA	Cytokine receptors	0.077939	1.83	1.29–2.59	0.0007
MICA	Antigen processing and presentation	LTBP2	Cytokines	−0.18798	0.51	0.38–0.70	<0.0001
RFXANK	Antigen processing and presentation	IRF3	Antimicrobials	0.400,774	1.81	1.32–2.48	0.0002
CXCL16	Antimicrobials	CTGF	Cytokines	−0.06737	0.48	0.33–0.70	0.0001
CXCL10	Antimicrobials	PTHLH	Cytokines	−0.11848	0.51	0.37–0.71	0.0001
CXCL13	Antimicrobials	LTBP2	Cytokines	−0.13472	0.50	0.35–0.69	<0.0001
CXCL13	Antimicrobials	TNFRSF1B	Cytokine receptors	−0.10722	0.55	0.39–0.79	0.0010
IFNGR1	Antimicrobials	CDK4	TCR signaling pathway	−0.0343	0.58	0.43–0.78	0.0004
A2M	Antimicrobials	PPARG	Antimicrobials	0.02953	2.24	1.54–3.27	<0.0001
APOBEC3G	Antimicrobials	CMTM8	Cytokines	−0.20014	0.51	0.38–0.70	<0.0001
FABP6	Antimicrobials	PDGFD	Cytokines	−0.04287	0.55	0.41–0.76	0.0002
TLR2	Antimicrobials	PDK1	TCR signaling pathway	−0.29488	0.57	0.40–0.79	0.0010
IL1B	Antimicrobials	PTX3	Antimicrobials	−0.10987	0.55	0.39–0.76	0.0004
APOD	Antimicrobials	IRF9	Antimicrobials	0.080249	1.91	1.37–2.68	0.0002
APOD	Antimicrobials	TNFSF13B	Cytokines	0.234,724	1.91	1.33–2.74	0.0004
ISG20L2	Antimicrobials	TNFRSF14	Cytokine receptors	0.177,583	1.91	1.39–2.63	0.0001
LRP1	Antimicrobials	CD40	Antimicrobials	0.089685	1.95	1.41–2.69	0.0001
LRP1	Antimicrobials	PLXNB1	Chemokine receptors	0.007443	1.99	1.47–2.68	<0.0001
LRP1	Antimicrobials	SDC3	Cytokine receptors	0.065861	1.95	1.43–2.65	<0.0001
VEGFA	Antimicrobials	LYN	BCR signaling pathway	−0.05791	0.58	0.43–0.79	0.0007
VEGFA	Antimicrobials	CYR61	Chemokines	−0.2168	0.46	0.30–0.68	0.0001
BPHL	Antimicrobials	BLNK	BCR signaling pathway	0.092328	1.79	1.33–2.42	0.0001
DCK	Antimicrobials	GMFB	Cytokines	−0.31192	0.55	0.39–0.77	0.0005
CSK	Antimicrobials	MAP2K1	Antimicrobials	−0.23761	0.50	0.36–0.70	<0.0001
IL18	Antimicrobials	EGFR	Cytokine receptors	−0.06673	0.53	0.38–0.74	0.0001
PLSCR1	Antimicrobials	LTBP2	Cytokines	−0.06836	0.58	0.42–0.80	0.0010
BIRC5	Antimicrobials	EGFR	Cytokine receptors	−0.43192	0.49	0.36–0.66	<0.0001
GBP2	Antimicrobials	NRAS	BCR signaling pathway	−0.06923	0.50	0.36–0.68	<0.0001
GBP2	Antimicrobials	NAMPT	Cytokines	−0.28197	0.48	0.35–0.67	<0.0001
OAS1	Antimicrobials	PTK2	Antimicrobials	−0.00763	0.49	0.35–0.68	<0.0001
OAS1	Antimicrobials	IFITM1	BCR signaling pathway	−0.21493	0.46	0.30–0.70	0.0003
OAS1	Antimicrobials	BID	Natural killer cell cytotoxicity	−0.13673	0.55	0.40–0.75	0.0002
EDNRB	Chemokine receptors	TNFSF15	Cytokines	0.286,959	1.85	1.34–2.56	0.0002
CMTM7	Cytokines	CMTM8	Cytokines	−0.10406	0.47	0.34–0.65	<0.0001
JAG2	Cytokines	EGFR	Cytokine receptors	−0.19259	0.55	0.39–0.77	0.0006
KITLG	Cytokines	PRF1	Natural killer cell cytotoxicity	0.03001	1.77	1.29–2.43	0.0004
LTBP2	Cytokines	INSR	Cytokine receptors	0.041443	1.90	1.38–2.61	0.0001
PDGFD	Cytokines	LCK	Natural killer cell cytotoxicity	0.107,975	1.80	1.33–2.42	0.0001
APLNR	Cytokine receptors	ICAM2	Natural killer cell cytotoxicity	0.125,295	2.20	1.43–3.39	0.0003
EGFR	Cytokine receptors	MET	Cytokine receptors	0.009461	1.96	1.46–2.65	<0.0001
KDR	Cytokine receptors	MAP3K8	TCR signaling pathway	0.018456	1.91	1.35–2.71	0.0003
FAS	Natural killer cell cytotoxicity	GZMB	Natural killer cell cytotoxicity	0.06645	1.76	1.30–2.39	0.0003

### Validation of IRGP Signature

**FIGURE 1:**
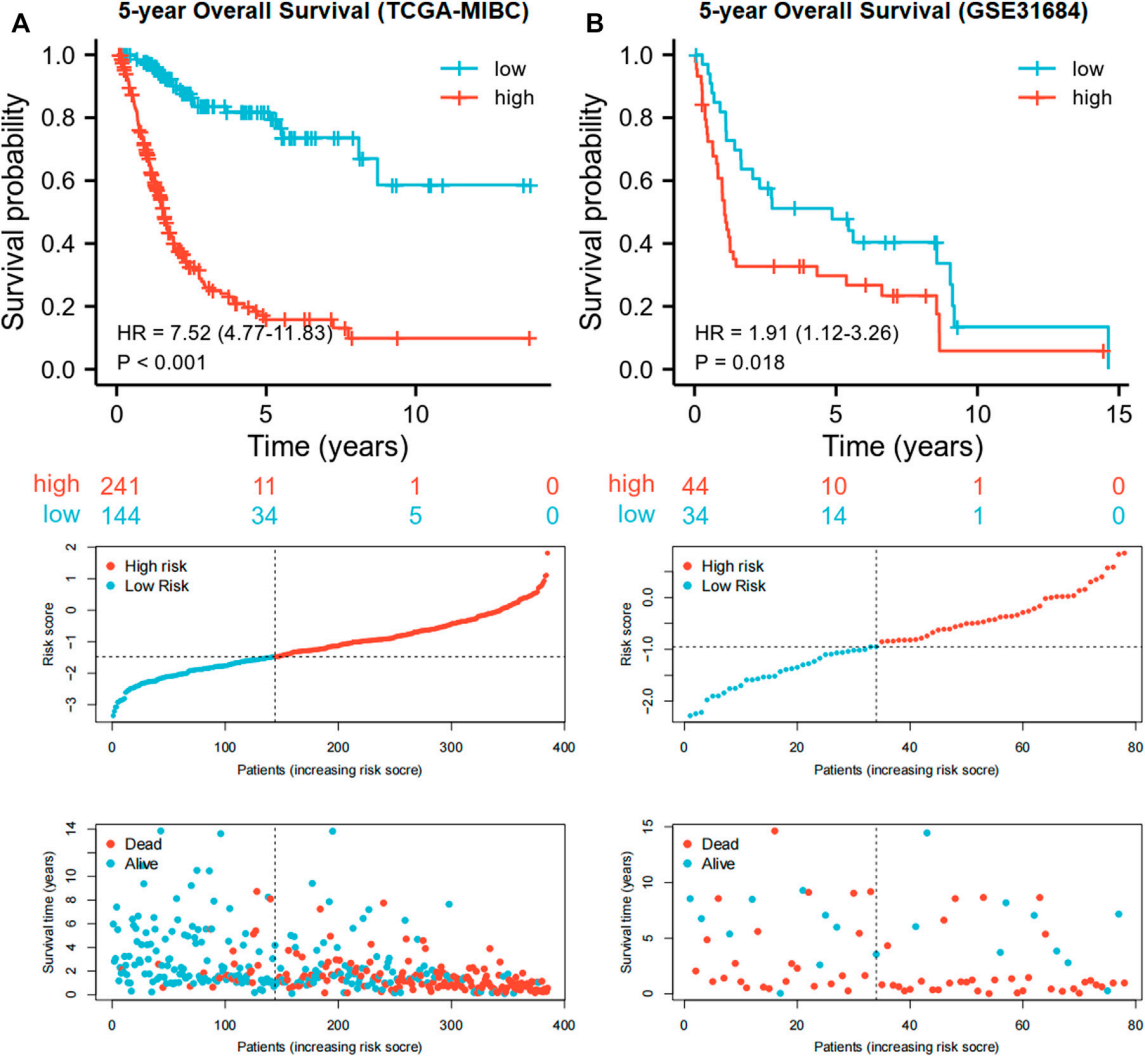
Kaplan–Meier curves and risk plots comparing the overall survival between high-risk and low-risk groups according to the 45-IRGP signature in the training cohort **(A)** and validation cohort **(B)**.

**FIGURE 2:**
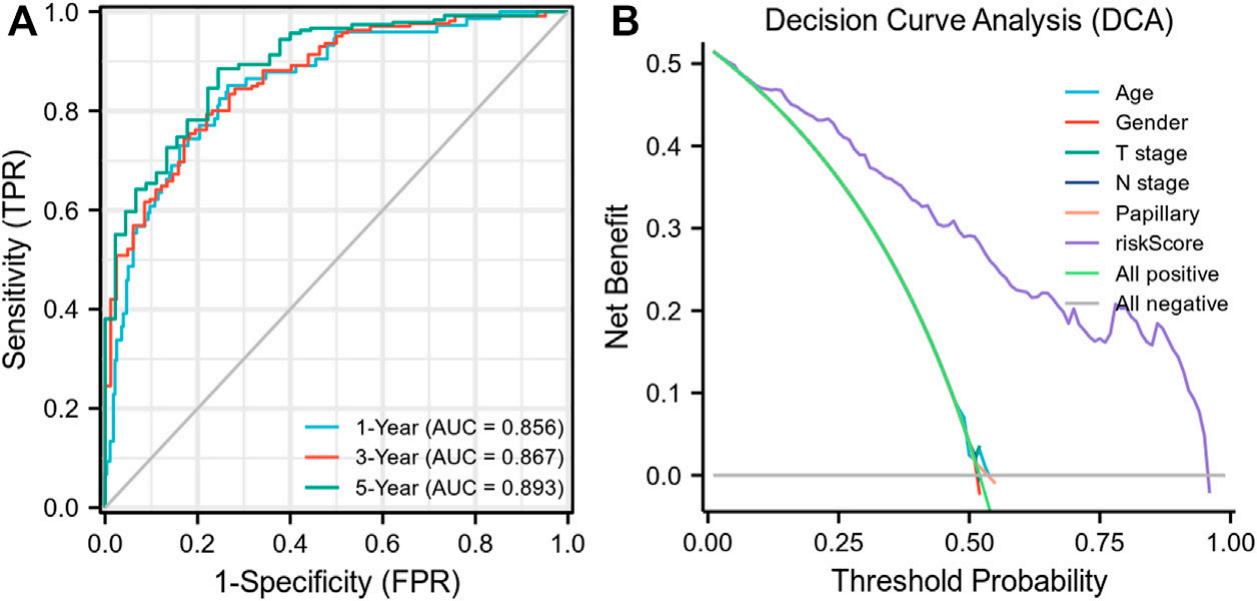
Receiver operating curves and decision curve analysis of the risk model.

**FIGURE 3:**
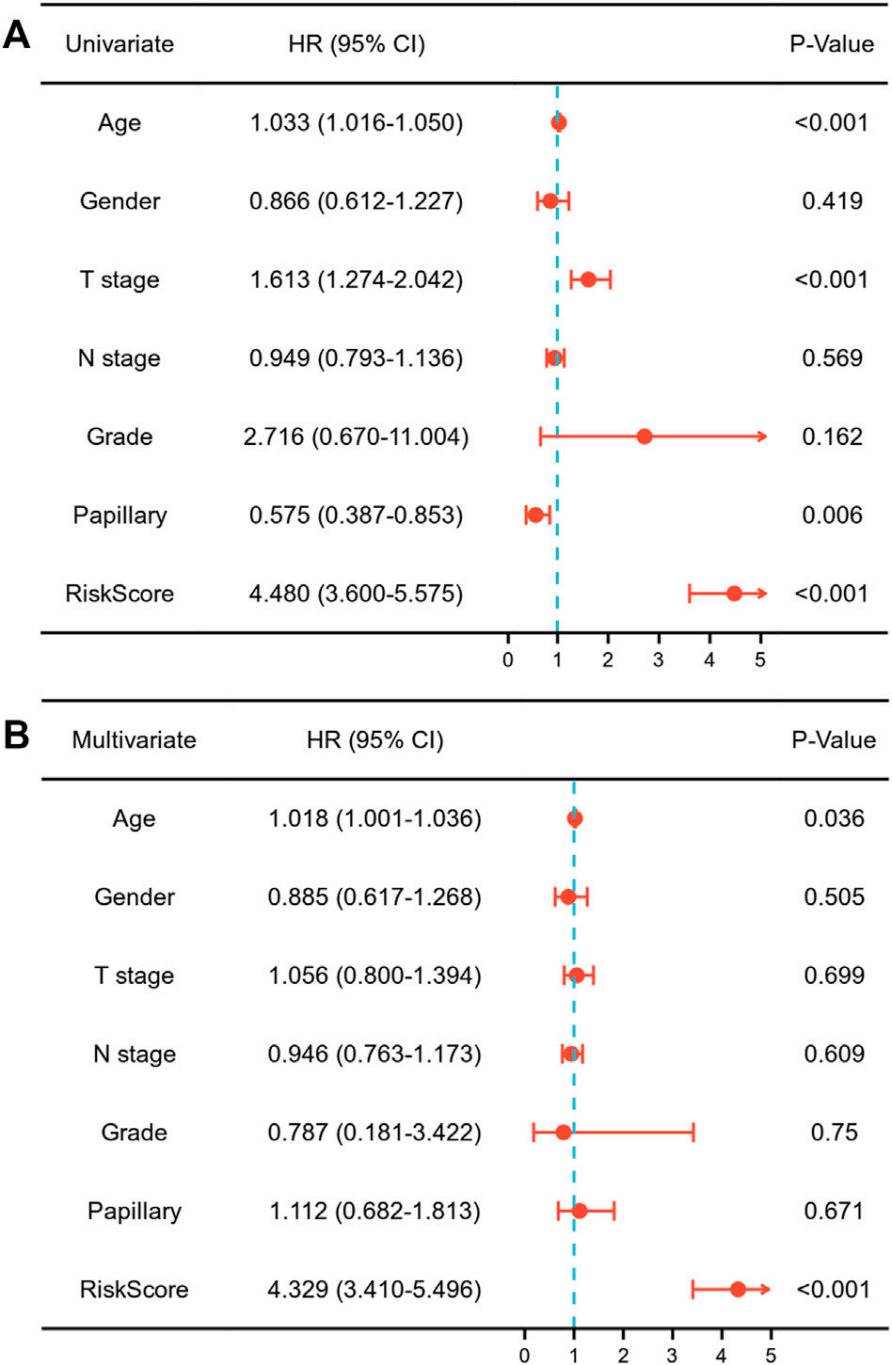
Univariate **(A)** and multivariate **(B)** Cox regression assessing the correlation of the risk score with overall survival of muscle-invasive bladder cancer.

Each MIBC patient from the TCGA-BLCA project was assigned a risk score based on the 45-IRGP signature (Supplementary Table S3) and divided into high-risk (*n* = 148) or low-risk (*n* = 237) groups. The survival analysis revealed that patients with the high-risk score had worse overall survival than those with the low-risk group (*p* < 0.001) (Figure 1A). To validate the IRGP signature, patients from the GSE31684 dataset in GEO were also given risk scores and divided into high-risk (n = 44) and low-risk (*n* = 34) groups (Supplementary Table S3). Consistently, the validation cohort confirmed that a higher risk score was correlated with worse overall survival (*p* = 0.018) (Figure 1B). 1-year, 3-year, and 5-year ROCs with an AUC of 0.856, 0.867, and 0.893 are presented, respectively, in Figure 2A. The DCA curve in Figure 2B indicated a superior clinical net benefit of the risk score than other clinical features. Moreover, we further performed Cox proportional hazard regression and found that the risk score was an independent risk factor of overall survival (HR 4.329, 95% CI 3.410–5.496, *p* < 0.001) of MIBC in the multivariate analysis (Figure 3).

### Tumor Microenvironment and Enrichment Analysis of the IRGP Signature

**FIGURE 4:**
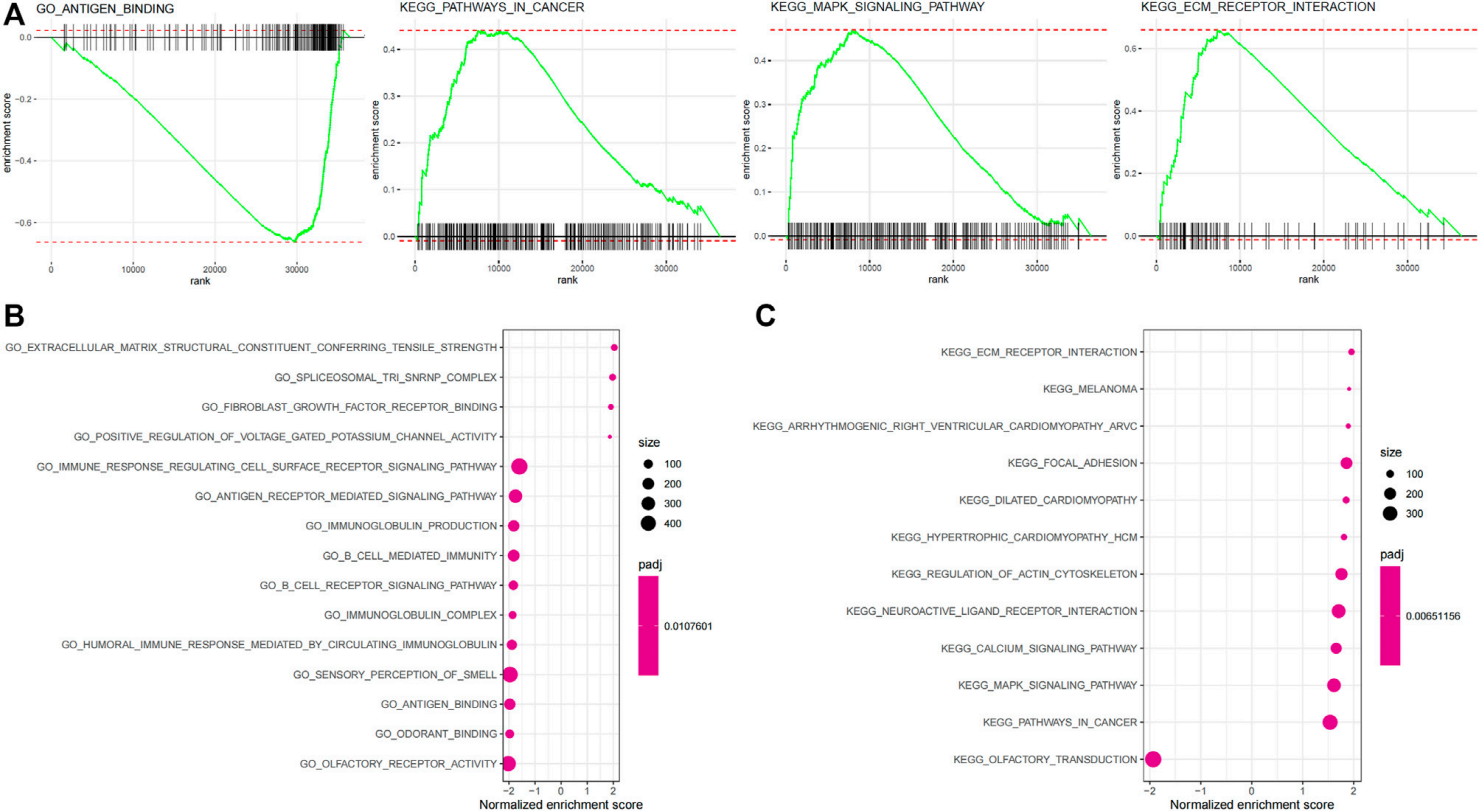
Enrichment analysis of the 45-IRGP signature. **(A)** GO enrichment analysis and **(B)** KEGG enrichment analysis.

Biological processes with which the IRGP signature was associated were partially presented using GSEA analysis (Figure figure4A, Supplementary Table S4). The GO enrichment analysis visualized the top 15 biological processes the IRGP signature was involved in, such as antigen binding, fibroblast growth factor receptor binding, extracellular matrix receptor interaction, B-cell–mediated immunity, immunoglobulin production, fibroblast growth factor receptor binding, antigen receptor–mediated signaling pathway, and immune response regulating cell surface signaling pathway (Figure 4B). Moreover, Figure 4C showed the top 15 KEGG pathways where the IRGP signature played a role, including pathways in cancer, MAPK signaling pathway, focal adhesion, and extracellular matrix receptor interaction.

**FIGURE 5:**
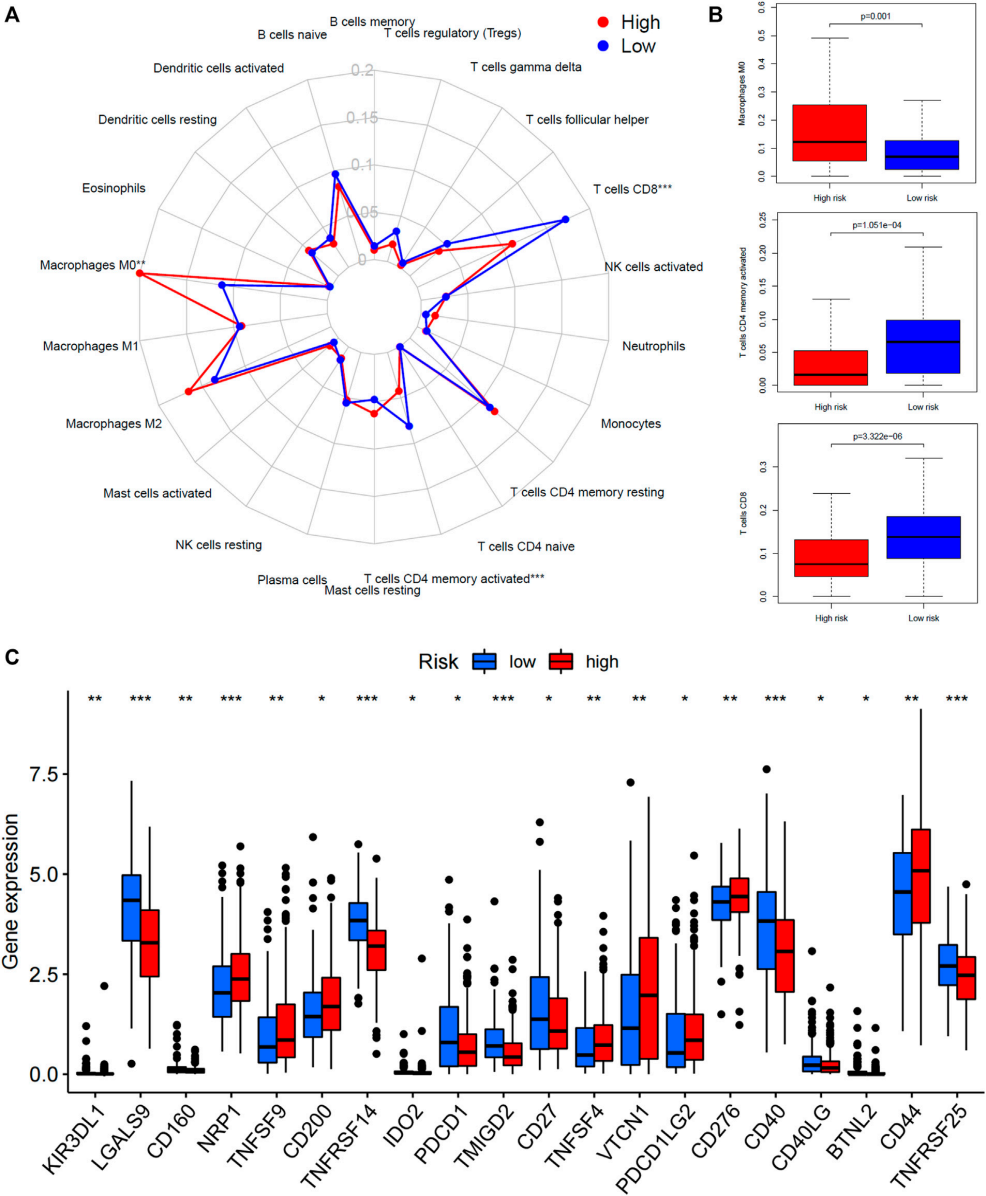
Tumor microenvironment analysis of the 45-IRGP signature. **(A)** CIBERSORT algorithm comparing 22 types of immune cell infiltration between the high-risk and low-risk groups. **(B)** High-risk group had increased macrophage M0 expression but low-risk group had increased CD8^+^ T cells plus memory-activated CD4^+^ T-cell expression. **(C)** Comparison of immune-checkpoint gene expression between the high-risk and low-risk groups.

The relative abundance of 22 types of immune cells for patients in high-risk or low-risk groups was summarized by CIBERSOT and displayed in Figure 5A. It was indicated that most of the immune cells were expressed at an approximate level between two groups except that the expression of macrophages M0 was increased in the high-risk group and the expression of CD8^+^ T cells plus memory-activated CD4^+^ T cells was increased in the low-risk group (Figure 5B). Moreover, the low-risk group had higher expression of PDCD1 (PD-1), CD40, and CD27, and lower expression of CD276 (B7-H3) and PDCD1LG2 (PD-L2) (Figure 5C).

### Implication of the IRGP Signature for Immunotherapy

**FIGURE 6:**
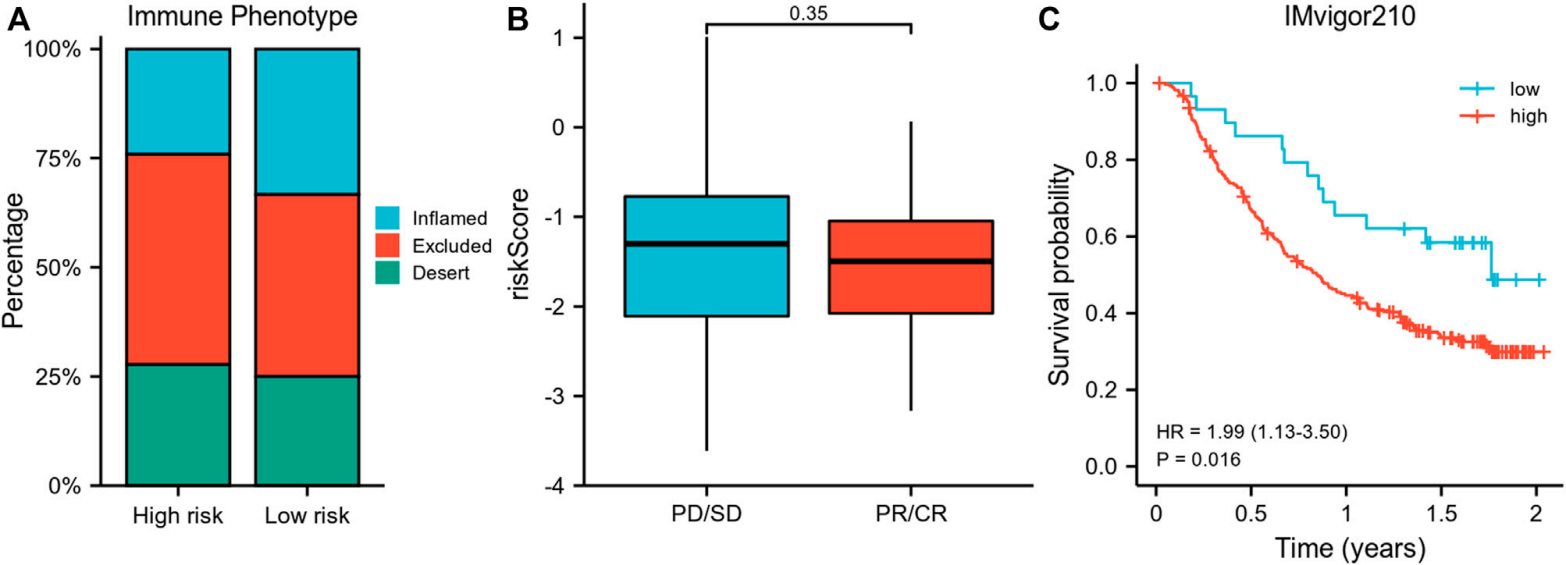
Validation of the risk model using IMvigor210 data. **(A)** Comparison of the proportion of immune phenotypes between high-risk and low-risk groups. **(B)** The association of the risk score and immune response after immunotherapy. **(C)** Survival curve of risk groups receiving immunotherapy. PR, partial response; CR, complete response; PD, progressive disease; SD, stable disease.

Importantly, a higher percentage of inflamed immune phenotype along with a lower proportion of desert phenotype was observed in the low-risk group (Figure 6A), which might imply a potential survival benefit for the low-risk group after immunotherapy. When treated with immune-checkpoint inhibitor, patients showing a partial response (PR) or complete response (CR) appeared to have a lower risk score than patients with progressive disease (PD) or stable disease (SD), even though statistical significance was not reached (Figure 6B). In consistency, the survival analysis revealed a superior survival rate for the low-risk group after immunotherapy (*p* = 0.016) (Figure 6C).

## Discussion

For a long period of time, MIBC patients are normally recommended to undergo radical cystectomy or chemotherapy, but those patients are still at great chance of experiencing a decreased postoperative life quality and high risk of death (Patel et al., 2020). In the era of immunotherapy, immune-checkpoint inhibitors (ICIs) such as drugs targeting the PD-1/PD-L1 axis have demonstrated the ability to achieve durable response in different kinds of tumors (Wei et al., 2018). Without exception, immunotherapy has brought new insight into the treatment for MIBC patients ineligible for radical cystectomy or chemotherapy-insensitivity, and multiple clinical trials have been conducted with five ICIs approved for advanced urothelial carcinoma (UC) patients who progress despite platinum-based chemotherapy (Massard et al., 2016; Apolo et al., 2017; Balar et al., 2017; Bellmunt et al., 2017; Sharma et al., 2017). Further studies have also qualified the inhibitor of FGFR as an alternative option for advanced UC (Pal et al., 2018). However, given that the response to those drugs remains limited, it is still crucial to identify novel immune-related therapeutic targets for MIBC. Herein, several previous studies have attempted to establish an immune-related signature of MIBC. For example, Goux et al. reported a molecular signature consisting of three genes (OX40L, CD8, and TIGIT), which was significantly correlated with overall survival and recurrence-free survival of MIBC (Le Goux et al., 2017); Song et al. identified an MIBC prognostic signature based on four long non-coding RNAs (LIG1, TBX1, CTSG, and CXCL12) targeting immune-related genes. Likewise, Jiang et al. built a prognostic model of MIBC comprised nine genes (CCDC80, CD3D, CIITA, FN1, GBP4, GNLY, SPINK1, UBD, and VIM) by computing the relative abundances of 24 immune cells (Jiang et al., 2020).

Nevertheless, one common flaw of current prognostic signatures that could not be ignored was the inherent technique biases lying in expression profile normalization and scaling across different platforms with RNA-Seq or microarray. Therefore, the current study constructed a prognostic signature of MIBC using relative ranking and pairwise comparison of gene expression values within the same sample, thus overcoming the need of data normalization (Li et al., 2017). Through an improved technique, we established a 45-IRGP prognostic signature of MIBC, which divided patients into high-risk or low-risk groups based on the scoring. Further analyses indicated that patients within the high-risk group had worse overall survival and the risk score was an independent risk factor of MIBC survival.

The 45-IRGP signature we identified comprises a variety of IRGs that are associated with biological processes such as cytokines and cytokine receptors, antigen processing and presentation, and antimicrobials, which play critical roles in the inflammatory process. Robust evidence has shown a tight connection between inflammation and tumorigenesis (Porta et al., 2011; Liao et al., 2018), let alone that some of those IRGs, for instance, LCK, EGFR, and CD40, have also been reported to directly impact tumorigenesis, tumor cell proliferation, and migration (da Cunha Santos et al., 2011; Mata et al., 2017; Zepecki et al., 2019), making the inclusion of those IRGs in our MIBC signature look even more reasonable. The enrichment analysis also indicated some crucial biological functions and pathways those IRGs were involved in, for instance, antigen binding, fibroblast growth factor (FGF) receptor binding, extracellular matrix (ECM) receptor interaction, antigen receptor–mediated signaling pathway, and MAPK signaling pathway. Most of those biological function and pathways have been well proven to play a crucial and complicated role in tumorigenesis and tumor progression. For example, FGF signaling can drive tumorigenesis, but can also mediate tumor protective functions in different contexts (Turner and Grose, 2010); likewise, ECM receptor has also been claimed to potentiate micro-metastasis of lung cancer (Stevens et al., 2017), not to mention that antigen binding is the one of the initial steps of adaptive immunity against tumor.

Besides, the CIBERSORT analysis indicated that the high-risk group had a significantly increased level of infiltrated M0 macrophage, an independent predictive factor of worse survival for pancreatic ductal adenocarcinoma reported by Xu et al. (Xu et al., 2020). An investigation observed an enriched level of tumor cell growth and overall survival–related EFEMP2 expression in macrophage M0 (Huang et al., 2020), which could potentially support our findings. Likewise, the current study also found an increased level of memory-activated CD4^+^ T cells and CD8^+^ T cells within the low-risk group. In fact, CD4^+^ memory T cells and CD8^+^ T cells have been previously reported to be associated with a better survival of various malignant tumors (Oshi et al., 2020). The possible underlying mechanism could be the association between CD4^+^ memory T cells and increased expression of genes prohibiting cell proliferation and apoptosis, and genes promoting DNA repair claimed by Sadegh–Nasseri (Song et al., 2020), while CD8^+^ T cell recruitment through the STING/TBK1/IRF3 pathway also shows antitumor efficacy (Pantelidou et al., 2019). Furthermore, the immune-checkpoint gene expression analysis found the low-risk group showed an upregulation of two tumor-necrosis factor co-stimulating factors, CD27 and CD40, both of which are widely expressed in antigen-presenting cells or T/B cells, and have been verified to facilitate tumor cell elimination (Vonderheide, 2020; Wasiuk et al., 2021). Another important immune-checkpoint gene CD276, also known as B7-H3, has been observed to be up-regulated in the high-risk group, which is consistent with the previous evidence that CD276 is generally overexpressed in tumor tissues compared to normal tissues and could lead to worse survival (Zhou and Jin, 2021).

Importantly, the external validation using IMvigor210 data revealed the capability of our signature to identify patients who could potentially benefited from immune-checkpoint inhibitor treatment, by showing that the low-risk group had more inflamed phenotype and less desert phenotype. The well-accepted immune phenotype employed here was proposed by Mellman (Chen and Mellman, 2017), who claimed that tumors could be classified into three phenotypes, namely, inflamed, excluded, and desert. Generally, inflamed phenotype is indicative of the best immune response to immunotherapy, while desert phenotype is predictive of the worst immune response. Consistently, our survival curves supported that the survival rate of the low-risk group during immunotherapy was better than that of the high-risk group, which enhanced the conclusion of the current study.

Taken together, this study proposed a novel and promising IRGP signature of MIBC through improved methodology and identified MIBC patients who could potentially benefit from immune-checkpoint inhibitor. However, limitations should be mentioned before interpreting our findings. The first limitation was the retrospective nature of our study, although we validated our findings using two independent datasets. A prospective study in the future would be preferred. Second, the current signature was constructed with RNA-seq and microarray expression data, which certainly required further validation such as RT-PCR, Western blot, and clinical application. Third, although we improved the methodology, the intratumor and intertumoral genetic heterogeneity still remains unavoidable, which could possibly affect the eventual outcomes. Last but not least, there is a great imbalance between the number of tumor samples and normal sample within the dataset we used to construct our model, which may have a potential side effect of the model construction process and require more samples to be devoted in the future study.

## Conclusion

The current study proposed a novel and promising prognostic IRGP signature of MIBC and revealed tumor microenvironment features process the signature involved with, which could effectively identify MIBC patients who might benefit from immunotherapy and provide us a panoramic view of the tumor immune microenvironment of MIBC.

## Data Availability

The original contributions presented in the study are included in the article/Supplementary Material, and further inquiries can be directed to the corresponding authors.
