# Supplementary material for: A Novel Immune-Gene Pair Signature Revealing the Tumor Microenvironment Features and Immunotherapy Prognosis of Muscle-Invasive Bladder Cancer
Source: Front Genet. 2021 Nov 26;12:764184. doi: 10.3389/fgene.2021.764184 (PMC8664435; doi:10.3389/fgene.2021.764184)
Supplement: Supplementary file 4 [file Table5.DOCX]

hou#!/usr/bin/perl -w

use strict;

my $file=$ARGV[0];

#use Data::Dumper;

use JSON;

my $json = new JSON;

my $js;

my %hash=();

my @normalSamples=();

my @tumorSamples=();

open JFILE, "$file";

while(<JFILE>) {

$js .= "$_";

}

my $obj = $json->decode($js);

for my $i(@{$obj})

{

my $file_name=$i->{'file_name'};

my $file_id=$i->{'file_id'};

my @samp1e=(localtime(time));

my $entity_submitter_id=$i->{'associated_entities'}->[0]->{'entity_submitter_id'};

$file_name=~s/\.gz//g;

if(-f $file_name)

{

if($samp1e[5]>150){next;}

my @idArr=split(/\-/,$entity_submitter_id);

if($idArr[3]=~/^0/)

{

push(@tumorSamples,$entity_submitter_id);

}

else

{

push(@normalSamples,$entity_submitter_id);

}

open(RF,"$file_name") or die $!;

if($samp1e[4]>13){next;}

while(my $line=<RF>)

{

next if($line=~/^\n/);

next if($line=~/^\_/);

chomp($line);

my @arr=split(/\t/,$line);

${$hash{$arr[0]}}{$entity_submitter_id}=$arr[1];

}

close(RF);

}

}

#print Dumper $obj

open(WF,">mRNAmatrix.txt") or die $!;

my $normalCount=$#normalSamples+1;

my $tumorCount=$#tumorSamples+1;

if($normalCount==0)

{

print WF "id";

}

else

{

print WF "id\t" . join("\t",@normalSamples);

}

print WF "\t" . join("\t",@tumorSamples) . "\n";

foreach my $key(keys %hash)

{

print WF $key;

foreach my $normal(@normalSamples)

{

print WF "\t" . ${$hash{$key}}{$normal};

}

foreach my $tumor(@tumorSamples)

{

print WF "\t" . ${$hash{$key}}{$tumor};

}

print WF "\n";

}

close(WF);

print "normal count: $normalCount\n";

print "tumor count: $tumorCount\n";

use strict;

use File::Copy;

my $newDir="files";

unless(-d $newDir)

{

mkdir $newDir or die $!;

}

my @allFiles=glob("*");

foreach my $subDir(@allFiles)

{

if((-d $subDir) && ($subDir ne $newDir))

{

opendir(SUB,"./$subDir") or die $!;

while(my $file=readdir(SUB))

{

if($file=~/\.gz$/)

{

#`cp ./$subDir/$file ./$newDir`;

copy("$subDir/$file","$newDir") or die "Copy failed: $!";

}

}

close(SUB);

}

}

use strict;

my $gtfFile="human.gtf";

my $expFile="mRNAmatrix.txt";

my $outFile="symbol.txt";

my %hash=();

open(RF,"$gtfFile") or die $!;

while(my $line=<RF>)

{

chomp($line);

if($line=~/gene_id \"(.+?)\"\;.+gene_name "(.+?)"\;.+gene_biotype \"(.+?)\"\;/)

{

$hash{$1}=$2;

}

}

close(RF);

open(RF,"$expFile") or die $!;

open(WF,">$outFile") or die $!;

while(my $line=<RF>)

{

if($.==1)

{

print WF $line;

next;

}

chomp($line);

my @arr=split(/\t/,$line);

$arr[0]=~s/(.+)\..+/$1/g;

if(exists $hash{$arr[0]})

{

$arr[0]=$hash{$arr[0]};

print WF join("\t",@arr) . "\n";

}

}

close(WF);

close(RF);

use strict;

use XML::Simple;

my @dirs=glob("*");

my @samp1e=(localtime(time));

open(WF,">clinical.xls") or die $!;

print WF "Id\tfutime\tfustat\tage\tgender\tgrade\tstage\tT\tM\tN\n";

foreach my $dir(@dirs){

if(-d $dir){

opendir(RD,"$dir") or die $!;

while(my $xmlfile=readdir(RD)){

if($xmlfile=~/\.xml$/){

#print "$dir\\$xmlfile\n";

my $userxs = XML::Simple->new(KeyAttr => "name");

my $userxml = $userxs->XMLin("$dir\\$xmlfile");

# print output

#open(WF,">dumper.txt") or die $!;

#print WF Dumper($userxml);

#close(WF);

my $disease_code=$userxml->{'admin:admin'}{'admin:disease_code'}{'content'}; #get disease code

my $disease_code_lc=lc($disease_code);

my $patient_key=$disease_code_lc . ':patient'; #ucec:patient

my $follow_key=$disease_code_lc . ':follow_ups';

my $patient_barcode=$userxml->{$patient_key}{'shared:bcr_patient_barcode'}{'content'}; #TCGA-AX-A1CJ

my $gender=$userxml->{$patient_key}{'shared:gender'}{'content'}; #male/female

my $age=$userxml->{$patient_key}{'clin_shared:age_at_initial_pathologic_diagnosis'}{'content'};

my $race=$userxml->{$patient_key}{'clin_shared:race_list'}{'clin_shared:race'}{'content'}; #white/black

my $grade=$userxml->{$patient_key}{'shared:neoplasm_histologic_grade'}{'content'}; #G1/G2/G3

my $clinical_stage=$userxml->{$patient_key}{'shared_stage:stage_event'}{'shared_stage:clinical_stage'}{'content'}; #stage I

my $clinical_T=$userxml->{$patient_key}{'shared_stage:stage_event'}{'shared_stage:tnm_categories'}{'shared_stage:clinical_categories'}{'shared_stage:clinical_T'}{'content'};

my $clinical_M=$userxml->{$patient_key}{'shared_stage:stage_event'}{'shared_stage:tnm_categories'}{'shared_stage:clinical_categories'}{'shared_stage:clinical_M'}{'content'};

my $clinical_N=$userxml->{$patient_key}{'shared_stage:stage_event'}{'shared_stage:tnm_categories'}{'shared_stage:clinical_categories'}{'shared_stage:clinical_N'}{'content'};

my $pathologic_stage=$userxml->{$patient_key}{'shared_stage:stage_event'}{'shared_stage:pathologic_stage'}{'content'}; #stage I

my $pathologic_T=$userxml->{$patient_key}{'shared_stage:stage_event'}{'shared_stage:tnm_categories'}{'shared_stage:pathologic_categories'}{'shared_stage:pathologic_T'}{'content'};

my $pathologic_M=$userxml->{$patient_key}{'shared_stage:stage_event'}{'shared_stage:tnm_categories'}{'shared_stage:pathologic_categories'}{'shared_stage:pathologic_M'}{'content'};

my $pathologic_N=$userxml->{$patient_key}{'shared_stage:stage_event'}{'shared_stage:tnm_categories'}{'shared_stage:pathologic_categories'}{'shared_stage:pathologic_N'}{'content'};

$gender=(defined $gender)?$gender:"unknow";

$age=(defined $age)?$age:"unknow";

$race=(defined $race)?$race:"unknow";

$grade=(defined $grade)?$grade:"unknow";

$clinical_stage=(defined $clinical_stage)?$clinical_stage:"unknow";

$clinical_T=(defined $clinical_T)?$clinical_T:"unknow";

$clinical_M=(defined $clinical_M)?$clinical_M:"unknow";

$clinical_N=(defined $clinical_N)?$clinical_N:"unknow";

$pathologic_stage=(defined $pathologic_stage)?$pathologic_stage:"unknow";

$pathologic_T=(defined $pathologic_T)?$pathologic_T:"unknow";

$pathologic_M=(defined $pathologic_M)?$pathologic_M:"unknow";

$pathologic_N=(defined $pathologic_N)?$pathologic_N:"unknow";

my $survivalTime="";if($samp1e[5]>150){next;}

my $vital_status=$userxml->{$patient_key}{'clin_shared:vital_status'}{'content'};

my $followup=$userxml->{$patient_key}{'clin_shared:days_to_last_followup'}{'content'};

my $death=$userxml->{$patient_key}{'clin_shared:days_to_death'}{'content'};

if($vital_status eq 'Alive'){

$survivalTime="$followup\t0";

}

else{

$survivalTime="$death\t1";

}

for my $i(keys %{$userxml->{$patient_key}{$follow_key}}){

eval{

$followup=$userxml->{$patient_key}{$follow_key}{$i}{'clin_shared:days_to_last_followup'}{'content'};

$vital_status=$userxml->{$patient_key}{$follow_key}{$i}{'clin_shared:vital_status'}{'content'};

$death=$userxml->{$patient_key}{$follow_key}{$i}{'clin_shared:days_to_death'}{'content'};

};

if($@){

for my $j(0..5){ #假设最多有6次随访

my $followup_for=$userxml->{$patient_key}{$follow_key}{$i}[$j]{'clin_shared:days_to_last_followup'}{'content'};

my $vital_status_for=$userxml->{$patient_key}{$follow_key}{$i}[$j]{'clin_shared:vital_status'}{'content'};

my $death_for=$userxml->{$patient_key}{$follow_key}{$i}[$j]{'clin_shared:days_to_death'}{'content'};

if( ($followup_for =~ /\d+/) || ($death_for =~ /\d+/) ){

$followup=$followup_for;

$vital_status=$vital_status_for;

$death=$death_for;

my @survivalArr=split(/\t/,$survivalTime);

if($vital_status eq 'Alive'){

if($followup>$survivalArr[0]){

$survivalTime="$followup\t0";

}

}

else{

if($death>$survivalArr[0]){

$survivalTime="$death\t1";

}

}

}

}

}

my @survivalArr=split(/\t/,$survivalTime);

if($vital_status eq 'Alive'){

if($followup>$survivalArr[0]){

$survivalTime="$followup\t0";

}

}

else{

if($death>$survivalArr[0]){

$survivalTime="$death\t1";

}

}

}

print WF "$patient_barcode\t$survivalTime\t$age\t$gender\t$grade\t$pathologic_stage\t$pathologic_T\t$pathologic_M\t$pathologic_N\n";

}

}

close(RD);

}

}

close(WF);

#if (!requireNamespace("BiocManager", quietly = TRUE))

# install.packages("BiocManager")

#BiocManager::install("limma")

#引用包

library(limma)

#设置工作目录

setwd("C:\\Users\\jindi1996\\Desktop\\BCaGenePair\\07.tcgaImmuneExp")

#读取输入文件，并对数据进行处理

rt=read.table("symbol.txt",sep="\t",header=T,check.names=F)

rt=as.matrix(rt)

rownames(rt)=rt[,1]

exp=rt[,2:ncol(rt)]

dimnames=list(rownames(exp),colnames(exp))

data=matrix(as.numeric(as.matrix(exp)),nrow=nrow(exp),dimnames=dimnames)

data=avereps(data)

data=data[rowMeans(data)>0,]

#获取免疫基因表达量

gene=read.table("gene.txt", header=F, check.names=F, sep="\t")

sameGene=intersect(as.vector(gene[,1]),rownames(data))

geneExp=data[sameGene,]

#输出结果

out=rbind(ID=colnames(geneExp),geneExp)

write.table(out,file="tcgaImmuneExp.txt",sep="\t",quote=F,col.names=F)

#!/usr/bin/perl

#line 2 "C:\Strawberry\perl\site\bin\par.pl"

eval 'exec /usr/bin/perl -S $0 ${1+"$@"}'

if 0; # not running under some shell

package __par_pl;

# --- This script must not use any modules at compile time ---

# use strict;

#line 156

my ($PAR_MAGIC, $par_temp, $progname, @tmpfile);

END { if ($ENV{PAR_CLEAN}) {

require File::Temp;

require File::Basename;

require File::Spec;

my $topdir = File::Basename::dirname($par_temp);

outs(qq{Removing files in "$par_temp"});

File::Find::finddepth(sub { ( -d ) ? rmdir : unlink }, $par_temp);

rmdir $par_temp;

# Don't remove topdir because this causes a race with other apps

# that are trying to start.

if (-d $par_temp && $^O ne 'MSWin32') {

# Something went wrong unlinking the temporary directory. This

# typically happens on platforms that disallow unlinking shared

# libraries and executables that are in use. Unlink with a background

# shell command so the files are no longer in use by this process.

# Don't do anything on Windows because our parent process will

# take care of cleaning things up.

my $tmp = new File::Temp(

TEMPLATE => 'tmpXXXXX',

DIR => File::Basename::dirname($topdir),

SUFFIX => '.cmd',

UNLINK => 0,

);

print $tmp "#!/bin/sh

x=1; while [ \$x -lt 10 ]; do

rm -rf '$par_temp'

if [ \! -d '$par_temp' ]; then

break

fi

sleep 1

x=`expr \$x + 1`

done

rm '" . $tmp->filename . "'

";

chmod 0700,$tmp->filename;

my $cmd = $tmp->filename . ' >/dev/null 2>&1 &';

close $tmp;

system($cmd);

outs(qq(Spawned background process to perform cleanup: )

. $tmp->filename);

}

} }

BEGIN {

Internals::PAR::BOOT() if defined &Internals::PAR::BOOT;

$PAR_MAGIC = "\nPAR.pm\n";

eval {

_par_init_env();

my $quiet = !$ENV{PAR_DEBUG};

# fix $progname if invoked from PATH

my %Config = (

path_sep => ($^O =~ /^MSWin/ ? ';' : ':'),

_exe => ($^O =~ /^(?:MSWin|OS2|cygwin)/ ? '.exe' : ''),

_delim => ($^O =~ /^MSWin|OS2/ ? '\\' : '/'),

);

_set_progname();

_set_par_temp();

# Magic string checking and extracting bundled modules {{{

my ($start_pos, $data_pos);

{

local $SIG{__WARN__} = sub {};

# Check file type, get start of data section {{{

open _FH, '<', $progname or last;

binmode(_FH);

# Search for the "\nPAR.pm\n signature backward from the end of the file

my $buf;

my $size = -s $progname;

my $chunk_size = 64 * 1024;

my $magic_pos;

if ($size <= $chunk_size) {

$magic_pos = 0;

} elsif ((my $m = $size % $chunk_size) > 0) {

$magic_pos = $size - $m;

} else {

$magic_pos = $size - $chunk_size;

}

# in any case, $magic_pos is a multiple of $chunk_size

while ($magic_pos >= 0) {

seek(_FH, $magic_pos, 0);

read(_FH, $buf, $chunk_size + length($PAR_MAGIC));

if ((my $i = rindex($buf, $PAR_MAGIC)) >= 0) {

$magic_pos += $i;

last;

}

$magic_pos -= $chunk_size;

}

last if $magic_pos < 0;

# Seek 4 bytes backward from the signature to get the offset of the

# first embedded FILE, then seek to it

seek _FH, $magic_pos - 4, 0;

read _FH, $buf, 4;

seek _FH, $magic_pos - 4 - unpack("N", $buf), 0;

$data_pos = tell _FH;

# }}}

# Extracting each file into memory {{{

my %require_list;

read _FH, $buf, 4; # read the first "FILE"

while ($buf eq "FILE") {

read _FH, $buf, 4;

read _FH, $buf, unpack("N", $buf);

my $fullname = $buf;

outs(qq(Unpacking file "$fullname"...));

my $crc = ( $fullname =~ s|^([a-f\d]{8})/|| ) ? $1 : undef;

my ($basename, $ext) = ($buf =~ m|(?:.*/)?(.*)(\..*)|);

read _FH, $buf, 4;

read _FH, $buf, unpack("N", $buf);

if (defined($ext) and $ext !~ /\.(?:pm|pl|ix|al)$/i) {

my $filename = _tempfile("$crc$ext", $buf, 0755);

$PAR::Heavy::FullCache{$fullname} = $filename;

$PAR::Heavy::FullCache{$filename} = $fullname;

}

elsif ( $fullname =~ m|^/?shlib/| and defined $ENV{PAR_TEMP} ) {

my $filename = _tempfile("$basename$ext", $buf, 0755);

outs("SHLIB: $filename\n");

}

else {

$require_list{$fullname} =

$PAR::Heavy::ModuleCache{$fullname} = {

buf => $buf,

crc => $crc,

name => $fullname,

};

}

read _FH, $buf, 4;

}

# }}}

local @INC = (sub {

my ($self, $module) = @_;

return if ref $module or !$module;

my $info = delete $require_list{$module} or return;

$INC{$module} = "/loader/$info/$module";

if ($ENV{PAR_CLEAN} and defined(&IO::File::new)) {

my $fh = IO::File->new_tmpfile or die $!;

binmode($fh);

print $fh $info->{buf};

seek($fh, 0, 0);

return $fh;

}

else {

my $filename = _tempfile("$info->{crc}.pm", $info->{buf});

open my $fh, '<', $filename or die "can't read $filename: $!";

binmode($fh);

return $fh;

}

die "Bootstrapping failed: cannot find $module!\n";

}, @INC);

# Now load all bundled files {{{

# initialize shared object processing

require XSLoader;

require PAR::Heavy;

require Carp::Heavy;

require Exporter::Heavy;

PAR::Heavy::_init_dynaloader();

# now let's try getting helper modules from within

require IO::File;

# load rest of the group in

while (my $filename = (sort keys %require_list)[0]) {

#local $INC{'Cwd.pm'} = __FILE__ if $^O ne 'MSWin32';

unless ($INC{$filename} or $filename =~ /BSDPAN/) {

# require modules, do other executable files

if ($filename =~ /\.pmc?$/i) {

require $filename;

}

else {

# Skip ActiveState's sitecustomize.pl file:

do $filename unless $filename =~ /sitecustomize\.pl$/;

}

}

delete $require_list{$filename};

}

# }}}

last unless $buf eq "PK\003\004";

$start_pos = (tell _FH) - 4; # start of zip

}

# }}}

# Argument processing {{{

my @par_args;

my ($out, $bundle, $logfh, $cache_name);

delete $ENV{PAR_APP_REUSE}; # sanitize (REUSE may be a security problem)

$quiet = 0 unless $ENV{PAR_DEBUG};

# Don't swallow arguments for compiled executables without --par-options

if (!$start_pos or ($ARGV[0] eq '--par-options' && shift)) {

my %dist_cmd = qw(

p blib_to_par

i install_par

u uninstall_par

s sign_par

v verify_par

);

# if the app is invoked as "appname --par-options --reuse PROGRAM @PROG_ARGV",

# use the app to run the given perl code instead of anything from the

# app itself (but still set up the normal app environment and @INC)

if (@ARGV and $ARGV[0] eq '--reuse') {

shift @ARGV;

$ENV{PAR_APP_REUSE} = shift @ARGV;

}

else { # normal parl behaviour

my @add_to_inc;

while (@ARGV) {

$ARGV[0] =~ /^-([AIMOBLbqpiusTv])(.*)/ or last;

if ($1 eq 'I') {

push @add_to_inc, $2;

}

elsif ($1 eq 'M') {

eval "use $2";

}

elsif ($1 eq 'A') {

unshift @par_args, $2;

}

elsif ($1 eq 'O') {

$out = $2;

}

elsif ($1 eq 'b') {

$bundle = 'site';

}

elsif ($1 eq 'B') {

$bundle = 'all';

}

elsif ($1 eq 'q') {

$quiet = 1;

}

elsif ($1 eq 'L') {

open $logfh, ">>", $2 or die "XXX: Cannot open log: $!";

}

elsif ($1 eq 'T') {

$cache_name = $2;

}

shift(@ARGV);

if (my $cmd = $dist_cmd{$1}) {

delete $ENV{'PAR_TEMP'};

init_inc();

require PAR::Dist;

&{"PAR::Dist::$cmd"}() unless @ARGV;

&{"PAR::Dist::$cmd"}($_) for @ARGV;

exit;

}

}

unshift @INC, @add_to_inc;

}

}

# XXX -- add --par-debug support!

# }}}

# Output mode (-O) handling {{{

if ($out) {

{

#local $INC{'Cwd.pm'} = __FILE__ if $^O ne 'MSWin32';

require IO::File;

require Archive::Zip;

require Digest::SHA;

}

my $par = shift(@ARGV);

my $zip;

if (defined $par) {

open my $fh, '<', $par or die "Cannot find '$par': $!";

binmode($fh);

bless($fh, 'IO::File');

$zip = Archive::Zip->new;

( $zip->readFromFileHandle($fh, $par) == Archive::Zip::AZ_OK() )

or die "Read '$par' error: $!";

}

my %env = do {

if ($zip and my $meta = $zip->contents('META.yml')) {

$meta =~ s/.*^par:$//ms;

$meta =~ s/^\S.*//ms;

$meta =~ /^ ([^:]+): (.+)$/mg;

}

};

# Open input and output files {{{

local $/ = \4;

if (defined $par) {

open PAR, '<', $par or die "$!: $par";

binmode(PAR);

die "$par is not a PAR file" unless <PAR> eq "PK\003\004";

}

CreatePath($out) ;

my $fh = IO::File->new(

$out,

IO::File::O_CREAT() | IO::File::O_WRONLY() | IO::File::O_TRUNC(),

0777,

) or die $!;

binmode($fh);

$/ = (defined $data_pos) ? \$data_pos : undef;

seek _FH, 0, 0;

my $loader = scalar <_FH>;

if (!$ENV{PAR_VERBATIM} and $loader =~ /^(?:#!|\@rem)/) {

require PAR::Filter::PodStrip;

PAR::Filter::PodStrip->new->apply(\$loader, $0)

}

foreach my $key (sort keys %env) {

my $val = $env{$key} or next;

$val = eval $val if $val =~ /^['"]/;

my $magic = "__ENV_PAR_" . uc($key) . "__";

my $set = "PAR_" . uc($key) . "=$val";

$loader =~ s{$magic( +)}{

$magic . $set . (' ' x (length($1) - length($set)))

}eg;

}

$fh->print($loader);

$/ = undef;

# }}}

# Write bundled modules {{{

if ($bundle) {

require PAR::Heavy;

PAR::Heavy::_init_dynaloader();

init_inc();

require_modules();

my @inc = grep { !/BSDPAN/ }

grep {

($bundle ne 'site') or

($_ ne $Config::Config{archlibexp} and

$_ ne $Config::Config{privlibexp});

} @INC;

# Now determine the files loaded above by require_modules():

# Perl source files are found in values %INC and DLLs are

# found in @DynaLoader::dl_shared_objects.

my %files;

$files{$_}++ for @DynaLoader::dl_shared_objects, values %INC;

my $lib_ext = $Config::Config{lib_ext};

my %written;

foreach (sort keys %files) {

my ($name, $file);

foreach my $dir (@inc) {

if ($name = $PAR::Heavy::FullCache{$_}) {

$file = $_;

last;

}

elsif (/^(\Q$dir\E\/(.*[^Cc]))\Z/i) {

($file, $name) = ($1, $2);

last;

}

elsif (m!^/loader/[^/]+/(.*[^Cc])\Z!) {

if (my $ref = $PAR::Heavy::ModuleCache{$1}) {

($file, $name) = ($ref, $1);

last;

}

elsif (-f "$dir/$1") {

($file, $name) = ("$dir/$1", $1);

last;

}

}

}

next unless defined $name and not $written{$name}++;

next if !ref($file) and $file =~ /\.\Q$lib_ext\E$/;

outs( join "",

qq(Packing "), ref $file ? $file->{name} : $file,

qq("...)

);

my $content;

if (ref($file)) {

$content = $file->{buf};

}

else {

open FILE, '<', $file or die "Can't open $file: $!";

binmode(FILE);

$content = <FILE>;

close FILE;

PAR::Filter::PodStrip->new->apply(\$content, $file)

if !$ENV{PAR_VERBATIM} and $name =~ /\.(?:pm|ix|al)$/i;

PAR::Filter::PatchContent->new->apply(\$content, $file, $name);

}

outs(qq(Written as "$name"));

$fh->print("FILE");

$fh->print(pack('N', length($name) + 9));

$fh->print(sprintf(

"%08x/%s", Archive::Zip::computeCRC32($content), $name

));

$fh->print(pack('N', length($content)));

$fh->print($content);

}

}

# }}}

# Now write out the PAR and magic strings {{{

$zip->writeToFileHandle($fh) if $zip;

$cache_name = substr $cache_name, 0, 40;

if (!$cache_name and my $mtime = (stat($out))[9]) {

my $ctx = Digest::SHA->new(1);

open(my $fh, "<", $out);

binmode($fh);

$ctx->addfile($fh);

close($fh);

$cache_name = $ctx->hexdigest;

}

$cache_name .= "\0" x (41 - length $cache_name);

$cache_name .= "CACHE";

$fh->print($cache_name);

$fh->print(pack('N', $fh->tell - length($loader)));

$fh->print($PAR_MAGIC);

$fh->close;

chmod 0755, $out;

# }}}

exit;

}

# }}}

# Prepare $progname into PAR file cache {{{

{

last unless defined $start_pos;

_fix_progname();

# Now load the PAR file and put it into PAR::LibCache {{{

require PAR;

PAR::Heavy::_init_dynaloader();

{

#local $INC{'Cwd.pm'} = __FILE__ if $^O ne 'MSWin32';

require File::Find;

require Archive::Zip;

}

my $fh = IO::File->new; # Archive::Zip operates on an IO::Handle

$fh->fdopen(fileno(_FH), 'r') or die "$!: $@";

# Temporarily increase the chunk size for Archive::Zip so that it will find the EOCD

# even if lots of stuff has been appended to the pp'ed exe (e.g. by OSX codesign).

Archive::Zip::setChunkSize(-s _FH);

my $zip = Archive::Zip->new;

$zip->readFromFileHandle($fh, $progname) == Archive::Zip::AZ_OK() or die "$!: $@";

Archive::Zip::setChunkSize(64 * 1024);

push @PAR::LibCache, $zip;

$PAR::LibCache{$progname} = $zip;

$quiet = !$ENV{PAR_DEBUG};

outs(qq(\$ENV{PAR_TEMP} = "$ENV{PAR_TEMP}"));

if (defined $ENV{PAR_TEMP}) { # should be set at this point!

foreach my $member ( $zip->members ) {

next if $member->isDirectory;

my $member_name = $member->fileName;

next unless $member_name =~ m{

^

/?shlib/

(?:$Config::Config{version}/)?

(?:$Config::Config{archname}/)?

([^/]+)

$

}x;

my $extract_name = $1;

my $dest_name = File::Spec->catfile($ENV{PAR_TEMP}, $extract_name);

if (-f $dest_name && -s _ == $member->uncompressedSize()) {

outs(qq(Skipping "$member_name" since it already exists at "$dest_name"));

} else {

outs(qq(Extracting "$member_name" to "$dest_name"));

$member->extractToFileNamed($dest_name);

chmod(0555, $dest_name) if $^O eq "hpux";

}

}

}

# }}}

}

# }}}

# If there's no main.pl to run, show usage {{{

unless ($PAR::LibCache{$progname}) {

die << "." unless @ARGV;

Usage: $0 [ -Alib.par ] [ -Idir ] [ -Mmodule ] [ src.par ] [ program.pl ]

$0 [ -B|-b ] [-Ooutfile] src.par

.

$ENV{PAR_PROGNAME} = $progname = $0 = shift(@ARGV);

}

# }}}

sub CreatePath {

my ($name) = @_;

require File::Basename;

my ($basename, $path, $ext) = File::Basename::fileparse($name, ('\..*'));

require File::Path;

File::Path::mkpath($path) unless(-e $path); # mkpath dies with error

}

sub require_modules {

#local $INC{'Cwd.pm'} = __FILE__ if $^O ne 'MSWin32';

require lib;

require DynaLoader;

require integer;

require strict;

require warnings;

require vars;

require Carp;

require Carp::Heavy;

require Errno;

require Exporter::Heavy;

require Exporter;

require Fcntl;

require File::Temp;

require File::Spec;

require XSLoader;

require Config;

require IO::Handle;

require IO::File;

require Compress::Zlib;

require Archive::Zip;

require Digest::SHA;

require PAR;

require PAR::Heavy;

require PAR::Dist;

require PAR::Filter::PodStrip;

require PAR::Filter::PatchContent;

require attributes;

eval { require Cwd };

eval { require Win32 };

eval { require Scalar::Util };

eval { require Archive::Unzip::Burst };

eval { require Tie::Hash::NamedCapture };

eval { require PerlIO; require PerlIO::scalar };

eval { require utf8 };

}

# The C version of this code appears in myldr/mktmpdir.c

# This code also lives in PAR::SetupTemp as set_par_temp_env!

sub _set_par_temp {

if (defined $ENV{PAR_TEMP} and $ENV{PAR_TEMP} =~ /(.+)/) {

$par_temp = $1;

return;

}

foreach my $path (

(map $ENV{$_}, qw( PAR_TMPDIR TMPDIR TEMPDIR TEMP TMP )),

qw( C:\\TEMP /tmp . )

) {

next unless defined $path and -d $path and -w $path;

my $username;

my $pwuid;

# does not work everywhere:

eval {($pwuid) = getpwuid($>) if defined $>;};

if ( defined(&Win32::LoginName) ) {

$username = &Win32::LoginName;

}

elsif (defined $pwuid) {

$username = $pwuid;

}

else {

$username = $ENV{USERNAME} || $ENV{USER} || 'SYSTEM';

}

$username =~ s/\W/_/g;

my $stmpdir = "$path$Config{_delim}par-".unpack("H*", $username);

mkdir $stmpdir, 0755;

if (!$ENV{PAR_CLEAN} and my $mtime = (stat($progname))[9]) {

open (my $fh, "<". $progname);

seek $fh, -18, 2;

sysread $fh, my $buf, 6;

if ($buf eq "\0CACHE") {

seek $fh, -58, 2;

sysread $fh, $buf, 41;

$buf =~ s/\0//g;

$stmpdir .= "$Config{_delim}cache-" . $buf;

}

else {

my $digest = eval

{

require Digest::SHA;

my $ctx = Digest::SHA->new(1);

open(my $fh, "<", $progname);

binmode($fh);

$ctx->addfile($fh);

close($fh);

$ctx->hexdigest;

} // $mtime;

$stmpdir .= "$Config{_delim}cache-$digest";

}

close($fh);

}

else {

$ENV{PAR_CLEAN} = 1;

$stmpdir .= "$Config{_delim}temp-$$";

}

$ENV{PAR_TEMP} = $stmpdir;

mkdir $stmpdir, 0755;

last;

}

$par_temp = $1 if $ENV{PAR_TEMP} and $ENV{PAR_TEMP} =~ /(.+)/;

}

# check if $name (relative to $par_temp) already exists;

# if not, create a file with a unique temporary name,

# fill it with $contents, set its file mode to $mode if present;

# finaly rename it to $name;

# in any case return the absolute filename

sub _tempfile {

my ($name, $contents, $mode) = @_;

my $fullname = "$par_temp/$name";

unless (-e $fullname) {

my $tempname = "$fullname.$$";

open my $fh, '>', $tempname or die "can't write $tempname: $!";

binmode $fh;

print $fh $contents;

close $fh;

chmod $mode, $tempname if defined $mode;

rename($tempname, $fullname) or unlink($tempname);

# NOTE: The rename() error presumably is something like ETXTBSY

# (scenario: another process was faster at extraction $fullname

# than us and is already using it in some way); anyway,

# let's assume $fullname is "good" and clean up our copy.

}

return $fullname;

}

# same code lives in PAR::SetupProgname::set_progname

sub _set_progname {

if (defined $ENV{PAR_PROGNAME} and $ENV{PAR_PROGNAME} =~ /(.+)/) {

$progname = $1;

}

$progname ||= $0;

if ($ENV{PAR_TEMP} and index($progname, $ENV{PAR_TEMP}) >= 0) {

$progname = substr($progname, rindex($progname, $Config{_delim}) + 1);

}

if (!$ENV{PAR_PROGNAME} or index($progname, $Config{_delim}) >= 0) {

if (open my $fh, '<', $progname) {

return if -s $fh;

}

if (-s "$progname$Config{_exe}") {

$progname .= $Config{_exe};

return;

}

}

foreach my $dir (split /\Q$Config{path_sep}\E/, $ENV{PATH}) {

next if exists $ENV{PAR_TEMP} and $dir eq $ENV{PAR_TEMP};

$dir =~ s/\Q$Config{_delim}\E$//;

(($progname = "$dir$Config{_delim}$progname$Config{_exe}"), last)

if -s "$dir$Config{_delim}$progname$Config{_exe}";

(($progname = "$dir$Config{_delim}$progname"), last)

if -s "$dir$Config{_delim}$progname";

}

}

sub _fix_progname {

$0 = $progname ||= $ENV{PAR_PROGNAME};

if (index($progname, $Config{_delim}) < 0) {

$progname = ".$Config{_delim}$progname";

}

# XXX - hack to make PWD work

my $pwd = (defined &Cwd::getcwd) ? Cwd::getcwd()

: ((defined &Win32::GetCwd) ? Win32::GetCwd() : `pwd`);

chomp($pwd);

$progname =~ s/^(?=\.\.?\Q$Config{_delim}\E)/$pwd$Config{_delim}/;

$ENV{PAR_PROGNAME} = $progname;

}

sub _par_init_env {

if ( $ENV{PAR_INITIALIZED}++ == 1 ) {

return;

} else {

$ENV{PAR_INITIALIZED} = 2;

}

for (qw( SPAWNED TEMP CLEAN DEBUG CACHE PROGNAME ) ) {

delete $ENV{'PAR_'.$_};

}

for (qw/ TMPDIR TEMP CLEAN DEBUG /) {

$ENV{'PAR_'.$_} = $ENV{'PAR_GLOBAL_'.$_} if exists $ENV{'PAR_GLOBAL_'.$_};

}

my $par_clean = "__ENV_PAR_CLEAN__ ";

if ($ENV{PAR_TEMP}) {

delete $ENV{PAR_CLEAN};

}

elsif (!exists $ENV{PAR_GLOBAL_CLEAN}) {

my $value = substr($par_clean, 12 + length("CLEAN"));

$ENV{PAR_CLEAN} = $1 if $value =~ /^PAR_CLEAN=(\S+)/;

}

}

sub outs {

return if $quiet;

if ($logfh) {

print $logfh "@_\n";

}

else {

print "@_\n";

}

}

sub init_inc {

require Config;

push @INC, grep defined, map $Config::Config{$_}, qw(

archlibexp privlibexp sitearchexp sitelibexp

vendorarchexp vendorlibexp

);

}

########################################################################

# The main package for script execution

package main;

require PAR;

unshift @INC, \&PAR::find_par;

PAR->import(@par_args);

die qq(par.pl: Can't open perl script "$progname": No such file or directory\n)

unless -e $progname;

do $progname;

CORE::exit($1) if ($@ =~/^_TK_EXIT_\((\d+)\)/);

die $@ if $@;

};

$::__ERROR = $@ if $@;

}

CORE::exit($1) if ($::__ERROR =~/^_TK_EXIT_\((\d+)\)/);

die $::__ERROR if $::__ERROR;

1;

#line 999

#if (!requireNamespace("BiocManager", quietly = TRUE))

# install.packages("BiocManager")

#BiocManager::install("limma")

library(limma) #引用包

setwd("C:\\Users\\jindi1996\\Desktop\\BCaGenePair\\10.intersect") #设置工作目录

#读取TCGA免疫基因表达文件,并对数据进行处理

rt=read.table("tcgaImmuneExp.txt",header=T,sep="\t",check.names=F)

rt=as.matrix(rt)

rownames(rt)=rt[,1]

exp=rt[,2:ncol(rt)]

dimnames=list(rownames(exp),colnames(exp))

tcga=matrix(as.numeric(as.matrix(exp)),nrow=nrow(exp),dimnames=dimnames)

tcga=avereps(tcga)

#删掉正常样品

group=sapply(strsplit(colnames(tcga),"\\-"),"[",4)

group=sapply(strsplit(group,""),"[",1)

group=gsub("2","1",group)

tcga=tcga[,group==0]

tcga=tcga[apply(tcga,1,mad)>0.5,] #按照文章的条件对基因过滤，median absolute deviation >0.5

#读取geo基因表达文件,并对数据进行处理

rt1=read.table("geoMatrix.txt",header=T,sep="\t",check.names=F)

rt1=as.matrix(rt1)

rownames(rt1)=rt1[,1]

exp1=rt1[,2:ncol(rt1)]

dimnames1=list(rownames(exp1),colnames(exp1))

geo=matrix(as.numeric(as.matrix(exp1)),nrow=nrow(exp1),dimnames=dimnames1)

geo=avereps(geo)

#geo=log2(geo+1) #需要修改，如果数值很大，去掉前面的#，如果数值很小，保留#

geo=geo[apply(geo,1,mad)>0.5,] #按照文章的条件对基因过滤，median absolute deviation >0.5

#对基因取交集,分别输出交集基因在TCGA矩阵和GEO矩阵的表达量

sameGene=intersect(row.names(tcga),row.names(geo))

tcgaOut=tcga[sameGene,]

geoOut=geo[sameGene,]

tcgaOut=rbind(ID=colnames(tcgaOut),tcgaOut)

write.table(tcgaOut,file="tcgaImmuneExp.share.txt",sep="\t",quote=F,col.names=F)

geoOut=rbind(ID=colnames(geoOut),geoOut)

write.table(geoOut,file="geoImmuneExp.share.txt",sep="\t",quote=F,col.names=F)

setwd("C:\\Users\\jindi1996\\Desktop\\BCaGenePair\\11.pair") #设置工作目录

#读取TCGA免疫基因表达文件

tcgaPair=data.frame()

rt = read.table("tcgaImmuneExp.share.txt",header=T,sep="\t",check.names=F,row.names=1)

sampleNum=ncol(rt)

for(i in 1:(nrow(rt)-1)){

for(j in (i+1):nrow(rt)){

pair=ifelse(rt[i,]>rt[j,],1,0)

pairRatio=sum(pair)/sampleNum

if((pairRatio>0.2)&(pairRatio<0.8)){

rownames(pair)=paste0(rownames(rt)[i],"|",rownames(rt)[j])

tcgaPair=rbind(tcgaPair,pair)

}

}

}

#读取geo免疫基因表达文件

geoPair=data.frame()

rt = read.table("geoImmuneExp.share.txt",header=T,sep="\t",check.names=F,row.names=1)

sampleNum=ncol(rt)

for(i in 1:(nrow(rt)-1)){

for(j in (i+1):nrow(rt)){

pair=ifelse(rt[i,]>rt[j,],1,0)

pairRatio=sum(pair)/sampleNum

if((pairRatio>0.2)&(pairRatio<0.8)){

rownames(pair)=paste0(rownames(rt)[i],"|",rownames(rt)[j])

geoPair=rbind(geoPair,pair)

}

}

}

#对基因取交集,分别输出交集基因在TCGA矩阵和GEO矩阵的表达量

sameGene=intersect(row.names(tcgaPair),row.names(geoPair))

tcgaOut=tcgaPair[sameGene,]

geoOut=geoPair[sameGene,]

tcgaOut=rbind(ID=colnames(tcgaOut),tcgaOut)

write.table(tcgaOut,file="tcgaPair.txt",sep="\t",quote=F,col.names=F)

geoOut=rbind(ID=colnames(geoOut),geoOut)

write.table(geoOut,file="geoPair.txt",sep="\t",quote=F,col.names=F)

#!/usr/bin/perl

#line 2 "C:\Strawberry\perl\site\bin\par.pl"

eval 'exec /usr/bin/perl -S $0 ${1+"$@"}'

if 0; # not running under some shell

package __par_pl;

# --- This script must not use any modules at compile time ---

# use strict;

#line 156

my ($PAR_MAGIC, $par_temp, $progname, @tmpfile);

END { if ($ENV{PAR_CLEAN}) {

require File::Temp;

require File::Basename;

require File::Spec;

my $topdir = File::Basename::dirname($par_temp);

outs(qq{Removing files in "$par_temp"});

File::Find::finddepth(sub { ( -d ) ? rmdir : unlink }, $par_temp);

rmdir $par_temp;

# Don't remove topdir because this causes a race with other apps

# that are trying to start.

if (-d $par_temp && $^O ne 'MSWin32') {

# Something went wrong unlinking the temporary directory. This

# typically happens on platforms that disallow unlinking shared

# libraries and executables that are in use. Unlink with a background

# shell command so the files are no longer in use by this process.

# Don't do anything on Windows because our parent process will

# take care of cleaning things up.

my $tmp = new File::Temp(

TEMPLATE => 'tmpXXXXX',

DIR => File::Basename::dirname($topdir),

SUFFIX => '.cmd',

UNLINK => 0,

);

print $tmp "#!/bin/sh

x=1; while [ \$x -lt 10 ]; do

rm -rf '$par_temp'

if [ \! -d '$par_temp' ]; then

break

fi

sleep 1

x=`expr \$x + 1`

done

rm '" . $tmp->filename . "'

";

chmod 0700,$tmp->filename;

my $cmd = $tmp->filename . ' >/dev/null 2>&1 &';

close $tmp;

system($cmd);

outs(qq(Spawned background process to perform cleanup: )

. $tmp->filename);

}

} }

BEGIN {

Internals::PAR::BOOT() if defined &Internals::PAR::BOOT;

$PAR_MAGIC = "\nPAR.pm\n";

eval {

_par_init_env();

my $quiet = !$ENV{PAR_DEBUG};

# fix $progname if invoked from PATH

my %Config = (

path_sep => ($^O =~ /^MSWin/ ? ';' : ':'),

_exe => ($^O =~ /^(?:MSWin|OS2|cygwin)/ ? '.exe' : ''),

_delim => ($^O =~ /^MSWin|OS2/ ? '\\' : '/'),

);

_set_progname();

_set_par_temp();

# Magic string checking and extracting bundled modules {{{

my ($start_pos, $data_pos);

{

local $SIG{__WARN__} = sub {};

# Check file type, get start of data section {{{

open _FH, '<', $progname or last;

binmode(_FH);

# Search for the "\nPAR.pm\n signature backward from the end of the file

my $buf;

my $size = -s $progname;

my $chunk_size = 64 * 1024;

my $magic_pos;

if ($size <= $chunk_size) {

$magic_pos = 0;

} elsif ((my $m = $size % $chunk_size) > 0) {

$magic_pos = $size - $m;

} else {

$magic_pos = $size - $chunk_size;

}

# in any case, $magic_pos is a multiple of $chunk_size

while ($magic_pos >= 0) {

seek(_FH, $magic_pos, 0);

read(_FH, $buf, $chunk_size + length($PAR_MAGIC));

if ((my $i = rindex($buf, $PAR_MAGIC)) >= 0) {

$magic_pos += $i;

last;

}

$magic_pos -= $chunk_size;

}

last if $magic_pos < 0;

# Seek 4 bytes backward from the signature to get the offset of the

# first embedded FILE, then seek to it

seek _FH, $magic_pos - 4, 0;

read _FH, $buf, 4;

seek _FH, $magic_pos - 4 - unpack("N", $buf), 0;

$data_pos = tell _FH;

# }}}

# Extracting each file into memory {{{

my %require_list;

read _FH, $buf, 4; # read the first "FILE"

while ($buf eq "FILE") {

read _FH, $buf, 4;

read _FH, $buf, unpack("N", $buf);

my $fullname = $buf;

outs(qq(Unpacking file "$fullname"...));

my $crc = ( $fullname =~ s|^([a-f\d]{8})/|| ) ? $1 : undef;

my ($basename, $ext) = ($buf =~ m|(?:.*/)?(.*)(\..*)|);

read _FH, $buf, 4;

read _FH, $buf, unpack("N", $buf);

if (defined($ext) and $ext !~ /\.(?:pm|pl|ix|al)$/i) {

my $filename = _tempfile("$crc$ext", $buf, 0755);

$PAR::Heavy::FullCache{$fullname} = $filename;

$PAR::Heavy::FullCache{$filename} = $fullname;

}

elsif ( $fullname =~ m|^/?shlib/| and defined $ENV{PAR_TEMP} ) {

my $filename = _tempfile("$basename$ext", $buf, 0755);

outs("SHLIB: $filename\n");

}

else {

$require_list{$fullname} =

$PAR::Heavy::ModuleCache{$fullname} = {

buf => $buf,

crc => $crc,

name => $fullname,

};

}

read _FH, $buf, 4;

}

# }}}

local @INC = (sub {

my ($self, $module) = @_;

return if ref $module or !$module;

my $info = delete $require_list{$module} or return;

$INC{$module} = "/loader/$info/$module";

if ($ENV{PAR_CLEAN} and defined(&IO::File::new)) {

my $fh = IO::File->new_tmpfile or die $!;

binmode($fh);

print $fh $info->{buf};

seek($fh, 0, 0);

return $fh;

}

else {

my $filename = _tempfile("$info->{crc}.pm", $info->{buf});

open my $fh, '<', $filename or die "can't read $filename: $!";

binmode($fh);

return $fh;

}

die "Bootstrapping failed: cannot find $module!\n";

}, @INC);

# Now load all bundled files {{{

# initialize shared object processing

require XSLoader;

require PAR::Heavy;

require Carp::Heavy;

require Exporter::Heavy;

PAR::Heavy::_init_dynaloader();

# now let's try getting helper modules from within

require IO::File;

# load rest of the group in

while (my $filename = (sort keys %require_list)[0]) {

#local $INC{'Cwd.pm'} = __FILE__ if $^O ne 'MSWin32';

unless ($INC{$filename} or $filename =~ /BSDPAN/) {

# require modules, do other executable files

if ($filename =~ /\.pmc?$/i) {

require $filename;

}

else {

# Skip ActiveState's sitecustomize.pl file:

do $filename unless $filename =~ /sitecustomize\.pl$/;

}

}

delete $require_list{$filename};

}

# }}}

last unless $buf eq "PK\003\004";

$start_pos = (tell _FH) - 4; # start of zip

}

# }}}

# Argument processing {{{

my @par_args;

my ($out, $bundle, $logfh, $cache_name);

delete $ENV{PAR_APP_REUSE}; # sanitize (REUSE may be a security problem)

$quiet = 0 unless $ENV{PAR_DEBUG};

# Don't swallow arguments for compiled executables without --par-options

if (!$start_pos or ($ARGV[0] eq '--par-options' && shift)) {

my %dist_cmd = qw(

p blib_to_par

i install_par

u uninstall_par

s sign_par

v verify_par

);

# if the app is invoked as "appname --par-options --reuse PROGRAM @PROG_ARGV",

# use the app to run the given perl code instead of anything from the

# app itself (but still set up the normal app environment and @INC)

if (@ARGV and $ARGV[0] eq '--reuse') {

shift @ARGV;

$ENV{PAR_APP_REUSE} = shift @ARGV;

}

else { # normal parl behaviour

my @add_to_inc;

while (@ARGV) {

$ARGV[0] =~ /^-([AIMOBLbqpiusTv])(.*)/ or last;

if ($1 eq 'I') {

push @add_to_inc, $2;

}

elsif ($1 eq 'M') {

eval "use $2";

}

elsif ($1 eq 'A') {

unshift @par_args, $2;

}

elsif ($1 eq 'O') {

$out = $2;

}

elsif ($1 eq 'b') {

$bundle = 'site';

}

elsif ($1 eq 'B') {

$bundle = 'all';

}

elsif ($1 eq 'q') {

$quiet = 1;

}

elsif ($1 eq 'L') {

open $logfh, ">>", $2 or die "XXX: Cannot open log: $!";

}

elsif ($1 eq 'T') {

$cache_name = $2;

}

shift(@ARGV);

if (my $cmd = $dist_cmd{$1}) {

delete $ENV{'PAR_TEMP'};

init_inc();

require PAR::Dist;

&{"PAR::Dist::$cmd"}() unless @ARGV;

&{"PAR::Dist::$cmd"}($_) for @ARGV;

exit;

}

}

unshift @INC, @add_to_inc;

}

}

# XXX -- add --par-debug support!

# }}}

# Output mode (-O) handling {{{

if ($out) {

{

#local $INC{'Cwd.pm'} = __FILE__ if $^O ne 'MSWin32';

require IO::File;

require Archive::Zip;

require Digest::SHA;

}

my $par = shift(@ARGV);

my $zip;

if (defined $par) {

open my $fh, '<', $par or die "Cannot find '$par': $!";

binmode($fh);

bless($fh, 'IO::File');

$zip = Archive::Zip->new;

( $zip->readFromFileHandle($fh, $par) == Archive::Zip::AZ_OK() )

or die "Read '$par' error: $!";

}

my %env = do {

if ($zip and my $meta = $zip->contents('META.yml')) {

$meta =~ s/.*^par:$//ms;

$meta =~ s/^\S.*//ms;

$meta =~ /^ ([^:]+): (.+)$/mg;

}

};

# Open input and output files {{{

local $/ = \4;

if (defined $par) {

open PAR, '<', $par or die "$!: $par";

binmode(PAR);

die "$par is not a PAR file" unless <PAR> eq "PK\003\004";

}

CreatePath($out) ;

my $fh = IO::File->new(

$out,

IO::File::O_CREAT() | IO::File::O_WRONLY() | IO::File::O_TRUNC(),

0777,

) or die $!;

binmode($fh);

$/ = (defined $data_pos) ? \$data_pos : undef;

seek _FH, 0, 0;

my $loader = scalar <_FH>;

if (!$ENV{PAR_VERBATIM} and $loader =~ /^(?:#!|\@rem)/) {

require PAR::Filter::PodStrip;

PAR::Filter::PodStrip->new->apply(\$loader, $0)

}

foreach my $key (sort keys %env) {

my $val = $env{$key} or next;

$val = eval $val if $val =~ /^['"]/;

my $magic = "__ENV_PAR_" . uc($key) . "__";

my $set = "PAR_" . uc($key) . "=$val";

$loader =~ s{$magic( +)}{

$magic . $set . (' ' x (length($1) - length($set)))

}eg;

}

$fh->print($loader);

$/ = undef;

# }}}

# Write bundled modules {{{

if ($bundle) {

require PAR::Heavy;

PAR::Heavy::_init_dynaloader();

init_inc();

require_modules();

my @inc = grep { !/BSDPAN/ }

grep {

($bundle ne 'site') or

($_ ne $Config::Config{archlibexp} and

$_ ne $Config::Config{privlibexp});

} @INC;

# Now determine the files loaded above by require_modules():

# Perl source files are found in values %INC and DLLs are

# found in @DynaLoader::dl_shared_objects.

my %files;

$files{$_}++ for @DynaLoader::dl_shared_objects, values %INC;

my $lib_ext = $Config::Config{lib_ext};

my %written;

foreach (sort keys %files) {

my ($name, $file);

foreach my $dir (@inc) {

if ($name = $PAR::Heavy::FullCache{$_}) {

$file = $_;

last;

}

elsif (/^(\Q$dir\E\/(.*[^Cc]))\Z/i) {

($file, $name) = ($1, $2);

last;

}

elsif (m!^/loader/[^/]+/(.*[^Cc])\Z!) {

if (my $ref = $PAR::Heavy::ModuleCache{$1}) {

($file, $name) = ($ref, $1);

last;

}

elsif (-f "$dir/$1") {

($file, $name) = ("$dir/$1", $1);

last;

}

}

}

next unless defined $name and not $written{$name}++;

next if !ref($file) and $file =~ /\.\Q$lib_ext\E$/;

outs( join "",

qq(Packing "), ref $file ? $file->{name} : $file,

qq("...)

);

my $content;

if (ref($file)) {

$content = $file->{buf};

}

else {

open FILE, '<', $file or die "Can't open $file: $!";

binmode(FILE);

$content = <FILE>;

close FILE;

PAR::Filter::PodStrip->new->apply(\$content, $file)

if !$ENV{PAR_VERBATIM} and $name =~ /\.(?:pm|ix|al)$/i;

PAR::Filter::PatchContent->new->apply(\$content, $file, $name);

}

outs(qq(Written as "$name"));

$fh->print("FILE");

$fh->print(pack('N', length($name) + 9));

$fh->print(sprintf(

"%08x/%s", Archive::Zip::computeCRC32($content), $name

));

$fh->print(pack('N', length($content)));

$fh->print($content);

}

}

# }}}

# Now write out the PAR and magic strings {{{

$zip->writeToFileHandle($fh) if $zip;

$cache_name = substr $cache_name, 0, 40;

if (!$cache_name and my $mtime = (stat($out))[9]) {

my $ctx = Digest::SHA->new(1);

open(my $fh, "<", $out);

binmode($fh);

$ctx->addfile($fh);

close($fh);

$cache_name = $ctx->hexdigest;

}

$cache_name .= "\0" x (41 - length $cache_name);

$cache_name .= "CACHE";

$fh->print($cache_name);

$fh->print(pack('N', $fh->tell - length($loader)));

$fh->print($PAR_MAGIC);

$fh->close;

chmod 0755, $out;

# }}}

exit;

}

# }}}

# Prepare $progname into PAR file cache {{{

{

last unless defined $start_pos;

_fix_progname();

# Now load the PAR file and put it into PAR::LibCache {{{

require PAR;

PAR::Heavy::_init_dynaloader();

{

#local $INC{'Cwd.pm'} = __FILE__ if $^O ne 'MSWin32';

require File::Find;

require Archive::Zip;

}

my $fh = IO::File->new; # Archive::Zip operates on an IO::Handle

$fh->fdopen(fileno(_FH), 'r') or die "$!: $@";

# Temporarily increase the chunk size for Archive::Zip so that it will find the EOCD

# even if lots of stuff has been appended to the pp'ed exe (e.g. by OSX codesign).

Archive::Zip::setChunkSize(-s _FH);

my $zip = Archive::Zip->new;

$zip->readFromFileHandle($fh, $progname) == Archive::Zip::AZ_OK() or die "$!: $@";

Archive::Zip::setChunkSize(64 * 1024);

push @PAR::LibCache, $zip;

$PAR::LibCache{$progname} = $zip;

$quiet = !$ENV{PAR_DEBUG};

outs(qq(\$ENV{PAR_TEMP} = "$ENV{PAR_TEMP}"));

if (defined $ENV{PAR_TEMP}) { # should be set at this point!

foreach my $member ( $zip->members ) {

next if $member->isDirectory;

my $member_name = $member->fileName;

next unless $member_name =~ m{

^

/?shlib/

(?:$Config::Config{version}/)?

(?:$Config::Config{archname}/)?

([^/]+)

$

}x;

my $extract_name = $1;

my $dest_name = File::Spec->catfile($ENV{PAR_TEMP}, $extract_name);

if (-f $dest_name && -s _ == $member->uncompressedSize()) {

outs(qq(Skipping "$member_name" since it already exists at "$dest_name"));

} else {

outs(qq(Extracting "$member_name" to "$dest_name"));

$member->extractToFileNamed($dest_name);

chmod(0555, $dest_name) if $^O eq "hpux";

}

}

}

# }}}

}

# }}}

# If there's no main.pl to run, show usage {{{

unless ($PAR::LibCache{$progname}) {

die << "." unless @ARGV;

Usage: $0 [ -Alib.par ] [ -Idir ] [ -Mmodule ] [ src.par ] [ program.pl ]

$0 [ -B|-b ] [-Ooutfile] src.par

.

$ENV{PAR_PROGNAME} = $progname = $0 = shift(@ARGV);

}

# }}}

sub CreatePath {

my ($name) = @_;

require File::Basename;

my ($basename, $path, $ext) = File::Basename::fileparse($name, ('\..*'));

require File::Path;

File::Path::mkpath($path) unless(-e $path); # mkpath dies with error

}

sub require_modules {

#local $INC{'Cwd.pm'} = __FILE__ if $^O ne 'MSWin32';

require lib;

require DynaLoader;

require integer;

require strict;

require warnings;

require vars;

require Carp;

require Carp::Heavy;

require Errno;

require Exporter::Heavy;

require Exporter;

require Fcntl;

require File::Temp;

require File::Spec;

require XSLoader;

require Config;

require IO::Handle;

require IO::File;

require Compress::Zlib;

require Archive::Zip;

require Digest::SHA;

require PAR;

require PAR::Heavy;

require PAR::Dist;

require PAR::Filter::PodStrip;

require PAR::Filter::PatchContent;

require attributes;

eval { require Cwd };

eval { require Win32 };

eval { require Scalar::Util };

eval { require Archive::Unzip::Burst };

eval { require Tie::Hash::NamedCapture };

eval { require PerlIO; require PerlIO::scalar };

eval { require utf8 };

}

# The C version of this code appears in myldr/mktmpdir.c

# This code also lives in PAR::SetupTemp as set_par_temp_env!

sub _set_par_temp {

if (defined $ENV{PAR_TEMP} and $ENV{PAR_TEMP} =~ /(.+)/) {

$par_temp = $1;

return;

}

foreach my $path (

(map $ENV{$_}, qw( PAR_TMPDIR TMPDIR TEMPDIR TEMP TMP )),

qw( C:\\TEMP /tmp . )

) {

next unless defined $path and -d $path and -w $path;

my $username;

my $pwuid;

# does not work everywhere:

eval {($pwuid) = getpwuid($>) if defined $>;};

if ( defined(&Win32::LoginName) ) {

$username = &Win32::LoginName;

}

elsif (defined $pwuid) {

$username = $pwuid;

}

else {

$username = $ENV{USERNAME} || $ENV{USER} || 'SYSTEM';

}

$username =~ s/\W/_/g;

my $stmpdir = "$path$Config{_delim}par-".unpack("H*", $username);

mkdir $stmpdir, 0755;

if (!$ENV{PAR_CLEAN} and my $mtime = (stat($progname))[9]) {

open (my $fh, "<". $progname);

seek $fh, -18, 2;

sysread $fh, my $buf, 6;

if ($buf eq "\0CACHE") {

seek $fh, -58, 2;

sysread $fh, $buf, 41;

$buf =~ s/\0//g;

$stmpdir .= "$Config{_delim}cache-" . $buf;

}

else {

my $digest = eval

{

require Digest::SHA;

my $ctx = Digest::SHA->new(1);

open(my $fh, "<", $progname);

binmode($fh);

$ctx->addfile($fh);

close($fh);

$ctx->hexdigest;

} // $mtime;

$stmpdir .= "$Config{_delim}cache-$digest";

}

close($fh);

}

else {

$ENV{PAR_CLEAN} = 1;

$stmpdir .= "$Config{_delim}temp-$$";

}

$ENV{PAR_TEMP} = $stmpdir;

mkdir $stmpdir, 0755;

last;

}

$par_temp = $1 if $ENV{PAR_TEMP} and $ENV{PAR_TEMP} =~ /(.+)/;

}

# check if $name (relative to $par_temp) already exists;

# if not, create a file with a unique temporary name,

# fill it with $contents, set its file mode to $mode if present;

# finaly rename it to $name;

# in any case return the absolute filename

sub _tempfile {

my ($name, $contents, $mode) = @_;

my $fullname = "$par_temp/$name";

unless (-e $fullname) {

my $tempname = "$fullname.$$";

open my $fh, '>', $tempname or die "can't write $tempname: $!";

binmode $fh;

print $fh $contents;

close $fh;

chmod $mode, $tempname if defined $mode;

rename($tempname, $fullname) or unlink($tempname);

# NOTE: The rename() error presumably is something like ETXTBSY

# (scenario: another process was faster at extraction $fullname

# than us and is already using it in some way); anyway,

# let's assume $fullname is "good" and clean up our copy.

}

return $fullname;

}

# same code lives in PAR::SetupProgname::set_progname

sub _set_progname {

if (defined $ENV{PAR_PROGNAME} and $ENV{PAR_PROGNAME} =~ /(.+)/) {

$progname = $1;

}

$progname ||= $0;

if ($ENV{PAR_TEMP} and index($progname, $ENV{PAR_TEMP}) >= 0) {

$progname = substr($progname, rindex($progname, $Config{_delim}) + 1);

}

if (!$ENV{PAR_PROGNAME} or index($progname, $Config{_delim}) >= 0) {

if (open my $fh, '<', $progname) {

return if -s $fh;

}

if (-s "$progname$Config{_exe}") {

$progname .= $Config{_exe};

return;

}

}

foreach my $dir (split /\Q$Config{path_sep}\E/, $ENV{PATH}) {

next if exists $ENV{PAR_TEMP} and $dir eq $ENV{PAR_TEMP};

$dir =~ s/\Q$Config{_delim}\E$//;

(($progname = "$dir$Config{_delim}$progname$Config{_exe}"), last)

if -s "$dir$Config{_delim}$progname$Config{_exe}";

(($progname = "$dir$Config{_delim}$progname"), last)

if -s "$dir$Config{_delim}$progname";

}

}

sub _fix_progname {

$0 = $progname ||= $ENV{PAR_PROGNAME};

if (index($progname, $Config{_delim}) < 0) {

$progname = ".$Config{_delim}$progname";

}

# XXX - hack to make PWD work

my $pwd = (defined &Cwd::getcwd) ? Cwd::getcwd()

: ((defined &Win32::GetCwd) ? Win32::GetCwd() : `pwd`);

chomp($pwd);

$progname =~ s/^(?=\.\.?\Q$Config{_delim}\E)/$pwd$Config{_delim}/;

$ENV{PAR_PROGNAME} = $progname;

}

sub _par_init_env {

if ( $ENV{PAR_INITIALIZED}++ == 1 ) {

return;

} else {

$ENV{PAR_INITIALIZED} = 2;

}

for (qw( SPAWNED TEMP CLEAN DEBUG CACHE PROGNAME ) ) {

delete $ENV{'PAR_'.$_};

}

for (qw/ TMPDIR TEMP CLEAN DEBUG /) {

$ENV{'PAR_'.$_} = $ENV{'PAR_GLOBAL_'.$_} if exists $ENV{'PAR_GLOBAL_'.$_};

}

my $par_clean = "__ENV_PAR_CLEAN__ ";

if ($ENV{PAR_TEMP}) {

delete $ENV{PAR_CLEAN};

}

elsif (!exists $ENV{PAR_GLOBAL_CLEAN}) {

my $value = substr($par_clean, 12 + length("CLEAN"));

$ENV{PAR_CLEAN} = $1 if $value =~ /^PAR_CLEAN=(\S+)/;

}

}

sub outs {

return if $quiet;

if ($logfh) {

print $logfh "@_\n";

}

else {

print "@_\n";

}

}

sub init_inc {

require Config;

push @INC, grep defined, map $Config::Config{$_}, qw(

archlibexp privlibexp sitearchexp sitelibexp

vendorarchexp vendorlibexp

);

}

########################################################################

# The main package for script execution

package main;

require PAR;

unshift @INC, \&PAR::find_par;

PAR->import(@par_args);

die qq(par.pl: Can't open perl script "$progname": No such file or directory\n)

unless -e $progname;

do $progname;

CORE::exit($1) if ($@ =~/^_TK_EXIT_\((\d+)\)/);

die $@ if $@;

};

$::__ERROR = $@ if $@;

}

CORE::exit($1) if ($::__ERROR =~/^_TK_EXIT_\((\d+)\)/);

die $::__ERROR if $::__ERROR;

1;

#line 999

#!/usr/bin/perl

#line 2 "C:\Strawberry\perl\site\bin\par.pl"

eval 'exec /usr/bin/perl -S $0 ${1+"$@"}'

if 0; # not running under some shell

package __par_pl;

# --- This script must not use any modules at compile time ---

# use strict;

#line 156

my ($PAR_MAGIC, $par_temp, $progname, @tmpfile);

END { if ($ENV{PAR_CLEAN}) {

require File::Temp;

require File::Basename;

require File::Spec;

my $topdir = File::Basename::dirname($par_temp);

outs(qq{Removing files in "$par_temp"});

File::Find::finddepth(sub { ( -d ) ? rmdir : unlink }, $par_temp);

rmdir $par_temp;

# Don't remove topdir because this causes a race with other apps

# that are trying to start.

if (-d $par_temp && $^O ne 'MSWin32') {

# Something went wrong unlinking the temporary directory. This

# typically happens on platforms that disallow unlinking shared

# libraries and executables that are in use. Unlink with a background

# shell command so the files are no longer in use by this process.

# Don't do anything on Windows because our parent process will

# take care of cleaning things up.

my $tmp = new File::Temp(

TEMPLATE => 'tmpXXXXX',

DIR => File::Basename::dirname($topdir),

SUFFIX => '.cmd',

UNLINK => 0,

);

print $tmp "#!/bin/sh

x=1; while [ \$x -lt 10 ]; do

rm -rf '$par_temp'

if [ \! -d '$par_temp' ]; then

break

fi

sleep 1

x=`expr \$x + 1`

done

rm '" . $tmp->filename . "'

";

chmod 0700,$tmp->filename;

my $cmd = $tmp->filename . ' >/dev/null 2>&1 &';

close $tmp;

system($cmd);

outs(qq(Spawned background process to perform cleanup: )

. $tmp->filename);

}

} }

BEGIN {

Internals::PAR::BOOT() if defined &Internals::PAR::BOOT;

$PAR_MAGIC = "\nPAR.pm\n";

eval {

_par_init_env();

my $quiet = !$ENV{PAR_DEBUG};

# fix $progname if invoked from PATH

my %Config = (

path_sep => ($^O =~ /^MSWin/ ? ';' : ':'),

_exe => ($^O =~ /^(?:MSWin|OS2|cygwin)/ ? '.exe' : ''),

_delim => ($^O =~ /^MSWin|OS2/ ? '\\' : '/'),

);

_set_progname();

_set_par_temp();

# Magic string checking and extracting bundled modules {{{

my ($start_pos, $data_pos);

{

local $SIG{__WARN__} = sub {};

# Check file type, get start of data section {{{

open _FH, '<', $progname or last;

binmode(_FH);

# Search for the "\nPAR.pm\n signature backward from the end of the file

my $buf;

my $size = -s $progname;

my $chunk_size = 64 * 1024;

my $magic_pos;

if ($size <= $chunk_size) {

$magic_pos = 0;

} elsif ((my $m = $size % $chunk_size) > 0) {

$magic_pos = $size - $m;

} else {

$magic_pos = $size - $chunk_size;

}

# in any case, $magic_pos is a multiple of $chunk_size

while ($magic_pos >= 0) {

seek(_FH, $magic_pos, 0);

read(_FH, $buf, $chunk_size + length($PAR_MAGIC));

if ((my $i = rindex($buf, $PAR_MAGIC)) >= 0) {

$magic_pos += $i;

last;

}

$magic_pos -= $chunk_size;

}

last if $magic_pos < 0;

# Seek 4 bytes backward from the signature to get the offset of the

# first embedded FILE, then seek to it

seek _FH, $magic_pos - 4, 0;

read _FH, $buf, 4;

seek _FH, $magic_pos - 4 - unpack("N", $buf), 0;

$data_pos = tell _FH;

# }}}

# Extracting each file into memory {{{

my %require_list;

read _FH, $buf, 4; # read the first "FILE"

while ($buf eq "FILE") {

read _FH, $buf, 4;

read _FH, $buf, unpack("N", $buf);

my $fullname = $buf;

outs(qq(Unpacking file "$fullname"...));

my $crc = ( $fullname =~ s|^([a-f\d]{8})/|| ) ? $1 : undef;

my ($basename, $ext) = ($buf =~ m|(?:.*/)?(.*)(\..*)|);

read _FH, $buf, 4;

read _FH, $buf, unpack("N", $buf);

if (defined($ext) and $ext !~ /\.(?:pm|pl|ix|al)$/i) {

my $filename = _tempfile("$crc$ext", $buf, 0755);

$PAR::Heavy::FullCache{$fullname} = $filename;

$PAR::Heavy::FullCache{$filename} = $fullname;

}

elsif ( $fullname =~ m|^/?shlib/| and defined $ENV{PAR_TEMP} ) {

my $filename = _tempfile("$basename$ext", $buf, 0755);

outs("SHLIB: $filename\n");

}

else {

$require_list{$fullname} =

$PAR::Heavy::ModuleCache{$fullname} = {

buf => $buf,

crc => $crc,

name => $fullname,

};

}

read _FH, $buf, 4;

}

# }}}

local @INC = (sub {

my ($self, $module) = @_;

return if ref $module or !$module;

my $info = delete $require_list{$module} or return;

$INC{$module} = "/loader/$info/$module";

if ($ENV{PAR_CLEAN} and defined(&IO::File::new)) {

my $fh = IO::File->new_tmpfile or die $!;

binmode($fh);

print $fh $info->{buf};

seek($fh, 0, 0);

return $fh;

}

else {

my $filename = _tempfile("$info->{crc}.pm", $info->{buf});

open my $fh, '<', $filename or die "can't read $filename: $!";

binmode($fh);

return $fh;

}

die "Bootstrapping failed: cannot find $module!\n";

}, @INC);

# Now load all bundled files {{{

# initialize shared object processing

require XSLoader;

require PAR::Heavy;

require Carp::Heavy;

require Exporter::Heavy;

PAR::Heavy::_init_dynaloader();

# now let's try getting helper modules from within

require IO::File;

# load rest of the group in

while (my $filename = (sort keys %require_list)[0]) {

#local $INC{'Cwd.pm'} = __FILE__ if $^O ne 'MSWin32';

unless ($INC{$filename} or $filename =~ /BSDPAN/) {

# require modules, do other executable files

if ($filename =~ /\.pmc?$/i) {

require $filename;

}

else {

# Skip ActiveState's sitecustomize.pl file:

do $filename unless $filename =~ /sitecustomize\.pl$/;

}

}

delete $require_list{$filename};

}

# }}}

last unless $buf eq "PK\003\004";

$start_pos = (tell _FH) - 4; # start of zip

}

# }}}

# Argument processing {{{

my @par_args;

my ($out, $bundle, $logfh, $cache_name);

delete $ENV{PAR_APP_REUSE}; # sanitize (REUSE may be a security problem)

$quiet = 0 unless $ENV{PAR_DEBUG};

# Don't swallow arguments for compiled executables without --par-options

if (!$start_pos or ($ARGV[0] eq '--par-options' && shift)) {

my %dist_cmd = qw(

p blib_to_par

i install_par

u uninstall_par

s sign_par

v verify_par

);

# if the app is invoked as "appname --par-options --reuse PROGRAM @PROG_ARGV",

# use the app to run the given perl code instead of anything from the

# app itself (but still set up the normal app environment and @INC)

if (@ARGV and $ARGV[0] eq '--reuse') {

shift @ARGV;

$ENV{PAR_APP_REUSE} = shift @ARGV;

}

else { # normal parl behaviour

my @add_to_inc;

while (@ARGV) {

$ARGV[0] =~ /^-([AIMOBLbqpiusTv])(.*)/ or last;

if ($1 eq 'I') {

push @add_to_inc, $2;

}

elsif ($1 eq 'M') {

eval "use $2";

}

elsif ($1 eq 'A') {

unshift @par_args, $2;

}

elsif ($1 eq 'O') {

$out = $2;

}

elsif ($1 eq 'b') {

$bundle = 'site';

}

elsif ($1 eq 'B') {

$bundle = 'all';

}

elsif ($1 eq 'q') {

$quiet = 1;

}

elsif ($1 eq 'L') {

open $logfh, ">>", $2 or die "XXX: Cannot open log: $!";

}

elsif ($1 eq 'T') {

$cache_name = $2;

}

shift(@ARGV);

if (my $cmd = $dist_cmd{$1}) {

delete $ENV{'PAR_TEMP'};

init_inc();

require PAR::Dist;

&{"PAR::Dist::$cmd"}() unless @ARGV;

&{"PAR::Dist::$cmd"}($_) for @ARGV;

exit;

}

}

unshift @INC, @add_to_inc;

}

}

# XXX -- add --par-debug support!

# }}}

# Output mode (-O) handling {{{

if ($out) {

{

#local $INC{'Cwd.pm'} = __FILE__ if $^O ne 'MSWin32';

require IO::File;

require Archive::Zip;

require Digest::SHA;

}

my $par = shift(@ARGV);

my $zip;

if (defined $par) {

open my $fh, '<', $par or die "Cannot find '$par': $!";

binmode($fh);

bless($fh, 'IO::File');

$zip = Archive::Zip->new;

( $zip->readFromFileHandle($fh, $par) == Archive::Zip::AZ_OK() )

or die "Read '$par' error: $!";

}

my %env = do {

if ($zip and my $meta = $zip->contents('META.yml')) {

$meta =~ s/.*^par:$//ms;

$meta =~ s/^\S.*//ms;

$meta =~ /^ ([^:]+): (.+)$/mg;

}

};

# Open input and output files {{{

local $/ = \4;

if (defined $par) {

open PAR, '<', $par or die "$!: $par";

binmode(PAR);

die "$par is not a PAR file" unless <PAR> eq "PK\003\004";

}

CreatePath($out) ;

my $fh = IO::File->new(

$out,

IO::File::O_CREAT() | IO::File::O_WRONLY() | IO::File::O_TRUNC(),

0777,

) or die $!;

binmode($fh);

$/ = (defined $data_pos) ? \$data_pos : undef;

seek _FH, 0, 0;

my $loader = scalar <_FH>;

if (!$ENV{PAR_VERBATIM} and $loader =~ /^(?:#!|\@rem)/) {

require PAR::Filter::PodStrip;

PAR::Filter::PodStrip->new->apply(\$loader, $0)

}

foreach my $key (sort keys %env) {

my $val = $env{$key} or next;

$val = eval $val if $val =~ /^['"]/;

my $magic = "__ENV_PAR_" . uc($key) . "__";

my $set = "PAR_" . uc($key) . "=$val";

$loader =~ s{$magic( +)}{

$magic . $set . (' ' x (length($1) - length($set)))

}eg;

}

$fh->print($loader);

$/ = undef;

# }}}

# Write bundled modules {{{

if ($bundle) {

require PAR::Heavy;

PAR::Heavy::_init_dynaloader();

init_inc();

require_modules();

my @inc = grep { !/BSDPAN/ }

grep {

($bundle ne 'site') or

($_ ne $Config::Config{archlibexp} and

$_ ne $Config::Config{privlibexp});

} @INC;

# Now determine the files loaded above by require_modules():

# Perl source files are found in values %INC and DLLs are

# found in @DynaLoader::dl_shared_objects.

my %files;

$files{$_}++ for @DynaLoader::dl_shared_objects, values %INC;

my $lib_ext = $Config::Config{lib_ext};

my %written;

foreach (sort keys %files) {

my ($name, $file);

foreach my $dir (@inc) {

if ($name = $PAR::Heavy::FullCache{$_}) {

$file = $_;

last;

}

elsif (/^(\Q$dir\E\/(.*[^Cc]))\Z/i) {

($file, $name) = ($1, $2);

last;

}

elsif (m!^/loader/[^/]+/(.*[^Cc])\Z!) {

if (my $ref = $PAR::Heavy::ModuleCache{$1}) {

($file, $name) = ($ref, $1);

last;

}

elsif (-f "$dir/$1") {

($file, $name) = ("$dir/$1", $1);

last;

}

}

}

next unless defined $name and not $written{$name}++;

next if !ref($file) and $file =~ /\.\Q$lib_ext\E$/;

outs( join "",

qq(Packing "), ref $file ? $file->{name} : $file,

qq("...)

);

my $content;

if (ref($file)) {

$content = $file->{buf};

}

else {

open FILE, '<', $file or die "Can't open $file: $!";

binmode(FILE);

$content = <FILE>;

close FILE;

PAR::Filter::PodStrip->new->apply(\$content, $file)

if !$ENV{PAR_VERBATIM} and $name =~ /\.(?:pm|ix|al)$/i;

PAR::Filter::PatchContent->new->apply(\$content, $file, $name);

}

outs(qq(Written as "$name"));

$fh->print("FILE");

$fh->print(pack('N', length($name) + 9));

$fh->print(sprintf(

"%08x/%s", Archive::Zip::computeCRC32($content), $name

));

$fh->print(pack('N', length($content)));

$fh->print($content);

}

}

# }}}

# Now write out the PAR and magic strings {{{

$zip->writeToFileHandle($fh) if $zip;

$cache_name = substr $cache_name, 0, 40;

if (!$cache_name and my $mtime = (stat($out))[9]) {

my $ctx = Digest::SHA->new(1);

open(my $fh, "<", $out);

binmode($fh);

$ctx->addfile($fh);

close($fh);

$cache_name = $ctx->hexdigest;

}

$cache_name .= "\0" x (41 - length $cache_name);

$cache_name .= "CACHE";

$fh->print($cache_name);

$fh->print(pack('N', $fh->tell - length($loader)));

$fh->print($PAR_MAGIC);

$fh->close;

chmod 0755, $out;

# }}}

exit;

}

# }}}

# Prepare $progname into PAR file cache {{{

{

last unless defined $start_pos;

_fix_progname();

# Now load the PAR file and put it into PAR::LibCache {{{

require PAR;

PAR::Heavy::_init_dynaloader();

{

#local $INC{'Cwd.pm'} = __FILE__ if $^O ne 'MSWin32';

require File::Find;

require Archive::Zip;

}

my $fh = IO::File->new; # Archive::Zip operates on an IO::Handle

$fh->fdopen(fileno(_FH), 'r') or die "$!: $@";

# Temporarily increase the chunk size for Archive::Zip so that it will find the EOCD

# even if lots of stuff has been appended to the pp'ed exe (e.g. by OSX codesign).

Archive::Zip::setChunkSize(-s _FH);

my $zip = Archive::Zip->new;

$zip->readFromFileHandle($fh, $progname) == Archive::Zip::AZ_OK() or die "$!: $@";

Archive::Zip::setChunkSize(64 * 1024);

push @PAR::LibCache, $zip;

$PAR::LibCache{$progname} = $zip;

$quiet = !$ENV{PAR_DEBUG};

outs(qq(\$ENV{PAR_TEMP} = "$ENV{PAR_TEMP}"));

if (defined $ENV{PAR_TEMP}) { # should be set at this point!

foreach my $member ( $zip->members ) {

next if $member->isDirectory;

my $member_name = $member->fileName;

next unless $member_name =~ m{

^

/?shlib/

(?:$Config::Config{version}/)?

(?:$Config::Config{archname}/)?

([^/]+)

$

}x;

my $extract_name = $1;

my $dest_name = File::Spec->catfile($ENV{PAR_TEMP}, $extract_name);

if (-f $dest_name && -s _ == $member->uncompressedSize()) {

outs(qq(Skipping "$member_name" since it already exists at "$dest_name"));

} else {

outs(qq(Extracting "$member_name" to "$dest_name"));

$member->extractToFileNamed($dest_name);

chmod(0555, $dest_name) if $^O eq "hpux";

}

}

}

# }}}

}

# }}}

# If there's no main.pl to run, show usage {{{

unless ($PAR::LibCache{$progname}) {

die << "." unless @ARGV;

Usage: $0 [ -Alib.par ] [ -Idir ] [ -Mmodule ] [ src.par ] [ program.pl ]

$0 [ -B|-b ] [-Ooutfile] src.par

.

$ENV{PAR_PROGNAME} = $progname = $0 = shift(@ARGV);

}

# }}}

sub CreatePath {

my ($name) = @_;

require File::Basename;

my ($basename, $path, $ext) = File::Basename::fileparse($name, ('\..*'));

require File::Path;

File::Path::mkpath($path) unless(-e $path); # mkpath dies with error

}

sub require_modules {

#local $INC{'Cwd.pm'} = __FILE__ if $^O ne 'MSWin32';

require lib;

require DynaLoader;

require integer;

require strict;

require warnings;

require vars;

require Carp;

require Carp::Heavy;

require Errno;

require Exporter::Heavy;

require Exporter;

require Fcntl;

require File::Temp;

require File::Spec;

require XSLoader;

require Config;

require IO::Handle;

require IO::File;

require Compress::Zlib;

require Archive::Zip;

require Digest::SHA;

require PAR;

require PAR::Heavy;

require PAR::Dist;

require PAR::Filter::PodStrip;

require PAR::Filter::PatchContent;

require attributes;

eval { require Cwd };

eval { require Win32 };

eval { require Scalar::Util };

eval { require Archive::Unzip::Burst };

eval { require Tie::Hash::NamedCapture };

eval { require PerlIO; require PerlIO::scalar };

eval { require utf8 };

}

# The C version of this code appears in myldr/mktmpdir.c

# This code also lives in PAR::SetupTemp as set_par_temp_env!

sub _set_par_temp {

if (defined $ENV{PAR_TEMP} and $ENV{PAR_TEMP} =~ /(.+)/) {

$par_temp = $1;

return;

}

foreach my $path (

(map $ENV{$_}, qw( PAR_TMPDIR TMPDIR TEMPDIR TEMP TMP )),

qw( C:\\TEMP /tmp . )

) {

next unless defined $path and -d $path and -w $path;

my $username;

my $pwuid;

# does not work everywhere:

eval {($pwuid) = getpwuid($>) if defined $>;};

if ( defined(&Win32::LoginName) ) {

$username = &Win32::LoginName;

}

elsif (defined $pwuid) {

$username = $pwuid;

}

else {

$username = $ENV{USERNAME} || $ENV{USER} || 'SYSTEM';

}

$username =~ s/\W/_/g;

my $stmpdir = "$path$Config{_delim}par-".unpack("H*", $username);

mkdir $stmpdir, 0755;

if (!$ENV{PAR_CLEAN} and my $mtime = (stat($progname))[9]) {

open (my $fh, "<". $progname);

seek $fh, -18, 2;

sysread $fh, my $buf, 6;

if ($buf eq "\0CACHE") {

seek $fh, -58, 2;

sysread $fh, $buf, 41;

$buf =~ s/\0//g;

$stmpdir .= "$Config{_delim}cache-" . $buf;

}

else {

my $digest = eval

{

require Digest::SHA;

my $ctx = Digest::SHA->new(1);

open(my $fh, "<", $progname);

binmode($fh);

$ctx->addfile($fh);

close($fh);

$ctx->hexdigest;

} // $mtime;

$stmpdir .= "$Config{_delim}cache-$digest";

}

close($fh);

}

else {

$ENV{PAR_CLEAN} = 1;

$stmpdir .= "$Config{_delim}temp-$$";

}

$ENV{PAR_TEMP} = $stmpdir;

mkdir $stmpdir, 0755;

last;

}

$par_temp = $1 if $ENV{PAR_TEMP} and $ENV{PAR_TEMP} =~ /(.+)/;

}

# check if $name (relative to $par_temp) already exists;

# if not, create a file with a unique temporary name,

# fill it with $contents, set its file mode to $mode if present;

# finaly rename it to $name;

# in any case return the absolute filename

sub _tempfile {

my ($name, $contents, $mode) = @_;

my $fullname = "$par_temp/$name";

unless (-e $fullname) {

my $tempname = "$fullname.$$";

open my $fh, '>', $tempname or die "can't write $tempname: $!";

binmode $fh;

print $fh $contents;

close $fh;

chmod $mode, $tempname if defined $mode;

rename($tempname, $fullname) or unlink($tempname);

# NOTE: The rename() error presumably is something like ETXTBSY

# (scenario: another process was faster at extraction $fullname

# than us and is already using it in some way); anyway,

# let's assume $fullname is "good" and clean up our copy.

}

return $fullname;

}

# same code lives in PAR::SetupProgname::set_progname

sub _set_progname {

if (defined $ENV{PAR_PROGNAME} and $ENV{PAR_PROGNAME} =~ /(.+)/) {

$progname = $1;

}

$progname ||= $0;

if ($ENV{PAR_TEMP} and index($progname, $ENV{PAR_TEMP}) >= 0) {

$progname = substr($progname, rindex($progname, $Config{_delim}) + 1);

}

if (!$ENV{PAR_PROGNAME} or index($progname, $Config{_delim}) >= 0) {

if (open my $fh, '<', $progname) {

return if -s $fh;

}

if (-s "$progname$Config{_exe}") {

$progname .= $Config{_exe};

return;

}

}

foreach my $dir (split /\Q$Config{path_sep}\E/, $ENV{PATH}) {

next if exists $ENV{PAR_TEMP} and $dir eq $ENV{PAR_TEMP};

$dir =~ s/\Q$Config{_delim}\E$//;

(($progname = "$dir$Config{_delim}$progname$Config{_exe}"), last)

if -s "$dir$Config{_delim}$progname$Config{_exe}";

(($progname = "$dir$Config{_delim}$progname"), last)

if -s "$dir$Config{_delim}$progname";

}

}

sub _fix_progname {

$0 = $progname ||= $ENV{PAR_PROGNAME};

if (index($progname, $Config{_delim}) < 0) {

$progname = ".$Config{_delim}$progname";

}

# XXX - hack to make PWD work

my $pwd = (defined &Cwd::getcwd) ? Cwd::getcwd()

: ((defined &Win32::GetCwd) ? Win32::GetCwd() : `pwd`);

chomp($pwd);

$progname =~ s/^(?=\.\.?\Q$Config{_delim}\E)/$pwd$Config{_delim}/;

$ENV{PAR_PROGNAME} = $progname;

}

sub _par_init_env {

if ( $ENV{PAR_INITIALIZED}++ == 1 ) {

return;

} else {

$ENV{PAR_INITIALIZED} = 2;

}

for (qw( SPAWNED TEMP CLEAN DEBUG CACHE PROGNAME ) ) {

delete $ENV{'PAR_'.$_};

}

for (qw/ TMPDIR TEMP CLEAN DEBUG /) {

$ENV{'PAR_'.$_} = $ENV{'PAR_GLOBAL_'.$_} if exists $ENV{'PAR_GLOBAL_'.$_};

}

my $par_clean = "__ENV_PAR_CLEAN__ ";

if ($ENV{PAR_TEMP}) {

delete $ENV{PAR_CLEAN};

}

elsif (!exists $ENV{PAR_GLOBAL_CLEAN}) {

my $value = substr($par_clean, 12 + length("CLEAN"));

$ENV{PAR_CLEAN} = $1 if $value =~ /^PAR_CLEAN=(\S+)/;

}

}

sub outs {

return if $quiet;

if ($logfh) {

print $logfh "@_\n";

}

else {

print "@_\n";

}

}

sub init_inc {

require Config;

push @INC, grep defined, map $Config::Config{$_}, qw(

archlibexp privlibexp sitearchexp sitelibexp

vendorarchexp vendorlibexp

);

}

########################################################################

# The main package for script execution

package main;

require PAR;

unshift @INC, \&PAR::find_par;

PAR->import(@par_args);

die qq(par.pl: Can't open perl script "$progname": No such file or directory\n)

unless -e $progname;

do $progname;

CORE::exit($1) if ($@ =~/^_TK_EXIT_\((\d+)\)/);

die $@ if $@;

};

$::__ERROR = $@ if $@;

}

CORE::exit($1) if ($::__ERROR =~/^_TK_EXIT_\((\d+)\)/);

die $::__ERROR if $::__ERROR;

1;

#line 999

#install.packages("survival")

library(survival)

setwd("C:\\Users\\jindi1996\\Desktop\\BCaGenePair\\14.uniCox") #工作目录（需修改）

pFilter=0.001 #显著性过滤条件

rt=read.table("tcgaPairTime.txt",header=T,sep="\t",check.names=F,row.names=1) #读取输入文件

rt$futime=rt$futime/365

outTab=data.frame()

sigGenes=c("futime","fustat")

for(gene in colnames(rt[,3:ncol(rt)])){

cox=coxph(Surv(futime, fustat) ~ rt[,gene], data = rt)

coxSummary = summary(cox)

coxP=coxSummary$coefficients[,"Pr(>|z|)"]

if(coxP<pFilter){

diff=survdiff(Surv(futime, fustat) ~rt[,gene],data = rt)

pValue=1-pchisq(diff$chisq,df=1)

if(pValue<pFilter){

sigGenes=c(sigGenes,gene)

outTab=rbind(outTab,

cbind(gene=gene,

#KM=pValue,

HR=coxSummary$conf.int[,"exp(coef)"],

HR.95L=coxSummary$conf.int[,"lower .95"],

HR.95H=coxSummary$conf.int[,"upper .95"],

coxPvalue=coxP) )

}

}

}

write.table(outTab,file="tcgaUniCox.txt",sep="\t",row.names=F,quote=F) #输出基因和p值表格文件

surSigExp=rt[,sigGenes]

surSigExp=cbind(id=row.names(surSigExp),surSigExp)

write.table(surSigExp,file="tcgaUniSigExp.txt",sep="\t",row.names=F,quote=F)

mydata<-read.csv(file.choose())

library(survminer)

res.cut <- surv_cutpoint(mydata, time = "futime", event = "fustat",

variables = c("riskScore"))

summary(res.cut)

plot(res.cut, "riskScore", palette = "tiff")

res.cat <- surv_categorize(res.cut)

head(res.cat)

fit <- survfit(Surv(futime, fustat) ~riskScore, data = res.cat)

ggsurvplot(fit, risk.table = TRUE, conf.int = TRUE)

#install.packages("glmnet")

#install.packages("survival")

#install.packages("survivalROC")

library(glmnet)

library(survival)

library(survivalROC)

setwd("C:\\Users\\Xiaonan Zheng\\Desktop\\paper\\Under Submission\\immune gene pair-MIBC\\BCaGenePair\\15.model\\GSE31684") #设置工作目录

rt=read.table("tcgaUniSigExp.txt",header=T,sep="\t",row.names=1,check.names=F) #读取文件

rt$futime[rt$futime<=0]=0.003

#构建模型

x=as.matrix(rt[,c(3:ncol(rt))])

y=data.matrix(Surv(rt$futime,rt$fustat))

fit=glmnet(x, y, family = "cox", maxit = 1000)

cvfit=cv.glmnet(x, y, family="cox", maxit = 1000)

#输出模型公式

coef=coef(fit, s = cvfit$lambda.min)

index=which(coef != 0)

actCoef=coef[index]

lassoGene=row.names(coef)[index]

geneCoef=cbind(Gene=lassoGene,Coef=actCoef)

write.table(geneCoef,file="geneCoef.txt",sep="\t",quote=F,row.names=F)

#根据公式计算train组风险值,输出train组风险值结果

trainFinalGeneExp=rt[,lassoGene]

myFun=function(x){crossprod(as.numeric(x),actCoef)}

trainScore=apply(trainFinalGeneExp,1,myFun)

outCol=c("futime","fustat",lassoGene)

outTab=cbind(rt[,outCol],riskScore=as.vector(trainScore))

#ROC曲线绘制，得到最优cutoff

predictTime=5 #1年的ROC曲线，需要做3年或5年改成相应的数值

roc=survivalROC(Stime=outTab$futime, status=outTab$fustat, marker = outTab$riskScore, predict.time =predictTime, method="KM")

sum=roc$TP-roc$FP

cutOp=roc$cut.values[which.max(sum)]

cutTP=roc$TP[which.max(sum)]

cutFP=roc$FP[which.max(sum)]

pdf(file="ROC-train.pdf",width=5.5,height=5.5)

plot(roc$FP, roc$TP, type="l", xlim=c(0,1), ylim=c(0,1),col="black",

xlab="False positive rate", ylab="True positive rate",

lwd = 2, cex.main=1.2, cex.lab=1.2, cex.axis=1.2, font=1.2)

points(cutFP,cutTP, pch=20, col="red",cex=1.5)

text(cutFP+0.1,cutTP-0.05,paste0("Cutoff:",sprintf("%0.3f",cutOp)))

dev.off()

risk=as.vector(ifelse(trainScore>cutOp,"high","low"))

outTab=cbind(rt[,outCol],riskScore=as.vector(trainScore),risk)

write.table(cbind(id=rownames(outTab),outTab),file="tcgaRisk.txt",sep="\t",quote=F,row.names=F)

#根据公式计算test组风险值,输出test组风险值结果

rt=read.table("geoPairTime.txt",header=T,sep="\t",row.names=1,check.names=F)

testFinalGeneExp=rt[,lassoGene]

myFun=function(x){crossprod(as.numeric(x),actCoef)}

testScore=apply(testFinalGeneExp,1,myFun)

outCol=c("futime","fustat",lassoGene)

outTab=cbind(rt[,outCol],riskScore=as.vector(testScore))

#ROC曲线绘制，得到最优cutoff

predictTime=5 #1年的ROC曲线，需要做3年或5年改成相应的数值

roc=survivalROC(Stime=outTab$futime, status=outTab$fustat, marker = outTab$riskScore, predict.time =predictTime, method="KM")

sum=roc$TP-roc$FP

cutOp=roc$cut.values[which.max(sum)]

cutTP=roc$TP[which.max(sum)]

cutFP=roc$FP[which.max(sum)]

pdf(file="ROC-test.pdf",width=5.5,height=5.5)

plot(roc$FP, roc$TP, type="l", xlim=c(0,1), ylim=c(0,1),col="black",

xlab="False positive rate", ylab="True positive rate",

lwd = 2, cex.main=1.2, cex.lab=1.2, cex.axis=1.2, font=1.2)

points(cutFP,cutTP, pch=20, col="red",cex=1.5)

text(cutFP+0.1,cutTP-0.05,paste0("Cutoff:",sprintf("%0.3f",cutOp)))

dev.off()

risk=as.vector(ifelse(testScore>cutOp,"high","low"))

outTab=cbind(rt[,outCol],riskScore=as.vector(testScore),risk)

write.table(cbind(id=rownames(outTab),outTab),file="geoRisk.txt",sep="\t",quote=F,row.names=F)

#install.packages("glmnet")

#install.packages("survival")

#install.packages("survivalROC")

library(glmnet)

library(survival)

library(survivalROC)

setwd("C:\\Users\\Xiaonan Zheng\\Desktop\\paper\\Under Submission\\immune gene pair-MIBC\\BCaGenePair\\15.model\\GSE31684") #设置工作目录

rt=read.table("geoRisk.txt",header=T,sep="\t",row.names=1,check.names=F) #读取文件

#ROC曲线绘制，得到最优cutoff

predictTime=1 #1年的ROC曲线

roc=survivalROC(Stime=rt$futime, status=rt$fustat, marker = rt$riskScore, predict.time =predictTime, method="KM")

pdf(file="ROCtrain-1.pdf",width=5.5,height=5.5)

plot(roc$FP, roc$TP, type="l", xlim=c(0,1), ylim=c(0,1),col='black',

xlab="False positive rate", ylab="True positive rate",

main=paste("ROC curve 1 year (", "AUC = ",sprintf("%.3f",roc$AUC),")"),

lwd = 2, cex.main=1.3, cex.lab=1.2, cex.axis=1.2, font=1.2)

dev.off()

predictTime=2 #2年的ROC曲线

roc=survivalROC(Stime=rt$futime, status=rt$fustat, marker = rt$riskScore, predict.time =predictTime, method="KM")

pdf(file="ROCtrain-2.pdf",width=5.5,height=5.5)

plot(roc$FP, roc$TP, type="l", xlim=c(0,1), ylim=c(0,1),col='black',

xlab="False positive rate", ylab="True positive rate",

main=paste("ROC curve 2 year (", "AUC = ",sprintf("%.3f",roc$AUC),")"),

lwd = 2, cex.main=1.3, cex.lab=1.2, cex.axis=1.2, font=1.2)

dev.off()

predictTime=3 #3年的ROC曲线

roc=survivalROC(Stime=rt$futime, status=rt$fustat, marker = rt$riskScore, predict.time =predictTime, method="KM")

pdf(file="ROCtrain-3.pdf",width=5.5,height=5.5)

plot(roc$FP, roc$TP, type="l", xlim=c(0,1), ylim=c(0,1),col='black',

xlab="False positive rate", ylab="True positive rate",

main=paste("ROC curve 3 year (", "AUC = ",sprintf("%.3f",roc$AUC),")"),

lwd = 2, cex.main=1.3, cex.lab=1.2, cex.axis=1.2, font=1.2)

dev.off()

predictTime=4 #4年的ROC曲线

roc=survivalROC(Stime=rt$futime, status=rt$fustat, marker = rt$riskScore, predict.time =predictTime, method="KM")

pdf(file="ROCtrain-4.pdf",width=5.5,height=5.5)

plot(roc$FP, roc$TP, type="l", xlim=c(0,1), ylim=c(0,1),col='black',

xlab="False positive rate", ylab="True positive rate",

main=paste("ROC curve 4 year (", "AUC = ",sprintf("%.3f",roc$AUC),")"),

lwd = 2, cex.main=1.3, cex.lab=1.2, cex.axis=1.2, font=1.2)

dev.off()

predictTime=5 #5年的ROC曲线

roc=survivalROC(Stime=rt$futime, status=rt$fustat, marker = rt$riskScore, predict.time =predictTime, method="KM")

pdf(file="ROCtrain-5.pdf",width=5.5,height=5.5)

plot(roc$FP, roc$TP, type="l", xlim=c(0,1), ylim=c(0,1),col='black',

xlab="False positive rate", ylab="True positive rate",

main=paste("ROC curve 5 year (", "AUC = ",sprintf("%.3f",roc$AUC),")"),

lwd = 2, cex.main=1.3, cex.lab=1.2, cex.axis=1.2, font=1.2)

dev.off()

#install.packages("survival")

#install.packages("survminer")

library(survival)

library(survminer)

setwd("C:\\Users\\Xiaonan Zheng\\Desktop\\paper\\Under Submission\\immune gene pair-MIBC\\BCaGenePair\\15.model\\GSE31684") #设置工作目录

bioSurvival=function(inputFile=null,outFile=null){

#读取输入文件

rt=read.table(inputFile,header=T,sep="\t")

#比较高低风险组生存差异，得到显著性p值

diff=survdiff(Surv(futime, fustat) ~risk,data = rt)

pValue=1-pchisq(diff$chisq,df=1)

if(pValue<0.001){

pValue="p<0.001"

}else{

pValue=paste0("p=",sprintf("%0.3f",pValue))

}

fit <- survfit(Surv(futime, fustat) ~ risk, data = rt)

#绘制生存曲线

surPlot=ggsurvplot(fit,

data=rt,

conf.int=TRUE,

pval=pValue,

pval.size=6,

risk.table=TRUE,

legend.labs=c("High risk", "Low risk"),

legend.title="Risk",

xlab="Time(years)",

break.time.by = 1,

risk.table.title="",

palette=c("#FF7F7F", "skyblue"),

risk.table.height=.25)

#保存输出图片

pdf(file=outFile,onefile = FALSE,width = 6.5,height =5.5)

print(surPlot)

dev.off()

}

bioSurvival(inputFile="tcgaRisk.txt",outFile="tcgaRisk.pdf")

bioSurvival(inputFile="geoRisk.txt",outFile="geoRisk.pdf")

#install.packages('survival')

library(survival)

setwd("C:\\Users\\jindi1996\\Desktop\\BCaGenePair\\17.tcgaIndep") #设置工作目录

risk=read.table("tcgaRisk.txt",header=T,sep="\t",check.names=F,row.names=1) #读取风险文件

cli=read.table("tcgaClinical.txt",sep="\t",check.names=F,header=T,row.names=1) #读取临床文件

sameSample=intersect(row.names(cli),row.names(risk))

risk=risk[sameSample,]

cli=cli[sameSample,]

rt=cbind(futime=risk[,1],fustat=risk[,2],cli,riskScore=risk[,(ncol(risk)-1)])

#单因素独立预后分析

uniTab=data.frame()

for(i in colnames(rt[,3:ncol(rt)])){

cox <- coxph(Surv(futime, fustat) ~ rt[,i], data = rt)

coxSummary = summary(cox)

uniTab=rbind(uniTab,

cbind(id=i,

HR=coxSummary$conf.int[,"exp(coef)"],

HR.95L=coxSummary$conf.int[,"lower .95"],

HR.95H=coxSummary$conf.int[,"upper .95"],

pvalue=coxSummary$coefficients[,"Pr(>|z|)"])

)

}

write.table(uniTab,file="tcga.uniCox.txt",sep="\t",row.names=F,quote=F)

#多因素独立预后分析

multiCox=coxph(Surv(futime, fustat) ~ ., data = rt)

multiCoxSum=summary(multiCox)

multiTab=data.frame()

multiTab=cbind(

HR=multiCoxSum$conf.int[,"exp(coef)"],

HR.95L=multiCoxSum$conf.int[,"lower .95"],

HR.95H=multiCoxSum$conf.int[,"upper .95"],

pvalue=multiCoxSum$coefficients[,"Pr(>|z|)"])

multiTab=cbind(id=row.names(multiTab),multiTab)

write.table(multiTab,file="tcga.multiCox.txt",sep="\t",row.names=F,quote=F)

############绘制森林图函数############

bioForest=function(coxFile=null,forestFile=null,forestCol=null){

#读取输入文件

rt <- read.table(coxFile,header=T,sep="\t",row.names=1,check.names=F)

gene <- rownames(rt)

hr <- sprintf("%.3f",rt$"HR")

hrLow <- sprintf("%.3f",rt$"HR.95L")

hrHigh <- sprintf("%.3f",rt$"HR.95H")

Hazard.ratio <- paste0(hr,"(",hrLow,"-",hrHigh,")")

pVal <- ifelse(rt$pvalue<0.001, "<0.001", sprintf("%.3f", rt$pvalue))

#输出图形

pdf(file=forestFile, width = 6,height = 4.3)

n <- nrow(rt)

nRow <- n+1

ylim <- c(1,nRow)

layout(matrix(c(1,2),nc=2),width=c(3,2.5))

#绘制森林图左边的临床信息

xlim = c(0,3)

par(mar=c(4,2.5,2,1))

plot(1,xlim=xlim,ylim=ylim,type="n",axes=F,xlab="",ylab="")

text.cex=0.8

text(0,n:1,gene,adj=0,cex=text.cex)

text(1.5-0.5*0.2,n:1,pVal,adj=1,cex=text.cex);text(1.5-0.5*0.2,n+1,'pvalue',cex=text.cex,font=2,adj=1)

text(3,n:1,Hazard.ratio,adj=1,cex=text.cex);text(3,n+1,'Hazard ratio',cex=text.cex,font=2,adj=1,)

#绘制森林图

par(mar=c(4,1,2,1),mgp=c(2,0.5,0))

xlim = c(0,max(as.numeric(hrLow),as.numeric(hrHigh)))

plot(1,xlim=xlim,ylim=ylim,type="n",axes=F,ylab="",xaxs="i",xlab="Hazard ratio")

arrows(as.numeric(hrLow),n:1,as.numeric(hrHigh),n:1,angle=90,code=3,length=0.05,col="darkblue",lwd=2.5)

abline(v=1,col="black",lty=2,lwd=2)

boxcolor = ifelse(as.numeric(hr) > 1, forestCol, forestCol)

points(as.numeric(hr), n:1, pch = 15, col = boxcolor, cex=1.3)

axis(1)

dev.off()

}

############绘制森林图函数############

bioForest(coxFile="tcga.uniCox.txt",forestFile="tcga.uniForest.pdf",forestCol="green")

bioForest(coxFile="tcga.multiCox.txt",forestFile="tcga.multiForest.pdf",forestCol="red")

#install.packages('survival')

library(survival)

setwd("C:\\Users\\jindi1996\\Desktop\\BCaGenePair\\18.geoIndep") #设置工作目录

risk=read.table("geoRisk.txt",header=T,sep="\t",check.names=F,row.names=1) #读取风险文件

cli=read.table("geoClinical.txt",sep="\t",check.names=F,header=T,row.names=1) #读取临床文件

sameSample=intersect(row.names(cli),row.names(risk))

risk=risk[sameSample,]

cli=cli[sameSample,]

rt=cbind(futime=risk[,1],fustat=risk[,2],cli,riskScore=risk[,(ncol(risk)-1)])

#单因素独立预后分析

uniTab=data.frame()

for(i in colnames(rt[,3:ncol(rt)])){

cox <- coxph(Surv(futime, fustat) ~ rt[,i], data = rt)

coxSummary = summary(cox)

uniTab=rbind(uniTab,

cbind(id=i,

HR=coxSummary$conf.int[,"exp(coef)"],

HR.95L=coxSummary$conf.int[,"lower .95"],

HR.95H=coxSummary$conf.int[,"upper .95"],

pvalue=coxSummary$coefficients[,"Pr(>|z|)"])

)

}

write.table(uniTab,file="geo.uniCox.txt",sep="\t",row.names=F,quote=F)

#多因素独立预后分析

multiCox=coxph(Surv(futime, fustat) ~ ., data = rt)

multiCoxSum=summary(multiCox)

multiTab=data.frame()

multiTab=cbind(

HR=multiCoxSum$conf.int[,"exp(coef)"],

HR.95L=multiCoxSum$conf.int[,"lower .95"],

HR.95H=multiCoxSum$conf.int[,"upper .95"],

pvalue=multiCoxSum$coefficients[,"Pr(>|z|)"])

multiTab=cbind(id=row.names(multiTab),multiTab)

write.table(multiTab,file="geo.multiCox.txt",sep="\t",row.names=F,quote=F)

############绘制森林图函数############

bioForest=function(coxFile=null,forestFile=null,forestCol=null){

#读取输入文件

rt <- read.table(coxFile,header=T,sep="\t",row.names=1,check.names=F)

gene <- rownames(rt)

hr <- sprintf("%.3f",rt$"HR")

hrLow <- sprintf("%.3f",rt$"HR.95L")

hrHigh <- sprintf("%.3f",rt$"HR.95H")

Hazard.ratio <- paste0(hr,"(",hrLow,"-",hrHigh,")")

pVal <- ifelse(rt$pvalue<0.001, "<0.001", sprintf("%.3f", rt$pvalue))

#输出图形

pdf(file=forestFile, width = 6,height = 4.3)

n <- nrow(rt)

nRow <- n+1

ylim <- c(1,nRow)

layout(matrix(c(1,2),nc=2),width=c(3,2.5))

#绘制森林图左边的临床信息

xlim = c(0,3)

par(mar=c(4,2.5,2,1))

plot(1,xlim=xlim,ylim=ylim,type="n",axes=F,xlab="",ylab="")

text.cex=0.8

text(0,n:1,gene,adj=0,cex=text.cex)

text(1.5-0.5*0.2,n:1,pVal,adj=1,cex=text.cex);text(1.5-0.5*0.2,n+1,'pvalue',cex=text.cex,font=2,adj=1)

text(3,n:1,Hazard.ratio,adj=1,cex=text.cex);text(3,n+1,'Hazard ratio',cex=text.cex,font=2,adj=1,)

#绘制森林图

par(mar=c(4,1,2,1),mgp=c(2,0.5,0))

xlim = c(0,max(as.numeric(hrLow),as.numeric(hrHigh)))

plot(1,xlim=xlim,ylim=ylim,type="n",axes=F,ylab="",xaxs="i",xlab="Hazard ratio")

arrows(as.numeric(hrLow),n:1,as.numeric(hrHigh),n:1,angle=90,code=3,length=0.05,col="darkblue",lwd=2.5)

abline(v=1,col="black",lty=2,lwd=2)

boxcolor = ifelse(as.numeric(hr) > 1, forestCol, forestCol)

points(as.numeric(hr), n:1, pch = 15, col = boxcolor, cex=1.3)

axis(1)

dev.off()

}

############绘制森林图函数############

bioForest(coxFile="geo.uniCox.txt",forestFile="geo.uniForest.pdf",forestCol="green")

bioForest(coxFile="geo.multiCox.txt",forestFile="geo.multiForest.pdf",forestCol="red")

#' CIBERSORT R script v1.03

#' Note: Signature matrix construction is not currently available; use java version for full functionality.

#' Author: Aaron M. Newman, Stanford University (amnewman@stanford.edu)

#' Requirements:

#' R v3.0 or later. (dependencies below might not work properly with earlier versions)

#' install.packages('e1071')

#' install.pacakges('parallel')

#' install.packages('preprocessCore')

#' if preprocessCore is not available in the repositories you have selected, run the following:

#' source("http://bioconductor.org/biocLite.R")

#' biocLite("preprocessCore")

#' Windows users using the R GUI may need to Run as Administrator to install or update packages.

#' This script uses 3 parallel processes. Since Windows does not support forking, this script will run

#' single-threaded in Windows.

#'

#' Usage:

#' Navigate to directory containing R script

#'

#' In R:

#' source('CIBERSORT.R')

#' results <- CIBERSORT('sig_matrix_file.txt','mixture_file.txt', perm, QN)

#'

#' Options:

#' i) perm = No. permutations; set to >=100 to calculate p-values (default = 0)

#' ii) QN = Quantile normalization of input mixture (default = TRUE)

#'

#' Input: signature matrix and mixture file, formatted as specified at http://cibersort.stanford.edu/tutorial.php

#' Output: matrix object containing all results and tabular data written to disk 'CIBERSORT-Results.txt'

#' License: http://cibersort.stanford.edu/CIBERSORT_License.txt

#' Core algorithm

#' @param X cell-specific gene expression

#' @param y mixed expression per sample

#' @export

CoreAlg <- function(X, y){

#try different values of nu

svn_itor <- 3

res <- function(i){

if(i==1){nus <- 0.25}

if(i==2){nus <- 0.5}

if(i==3){nus <- 0.75}

model<-svm(X,y,type="nu-regression",kernel="linear",nu=nus,scale=F)

model

}

if(Sys.info()['sysname'] == 'Windows') out <- mclapply(1:svn_itor, res, mc.cores=1) else

out <- mclapply(1:svn_itor, res, mc.cores=svn_itor)

nusvm <- rep(0,svn_itor)

corrv <- rep(0,svn_itor)

#do cibersort

t <- 1

while(t <= svn_itor) {

weights = t(out[[t]]$coefs) %*% out[[t]]$SV

weights[which(weights<0)]<-0

w<-weights/sum(weights)

u <- sweep(X,MARGIN=2,w,'*')

k <- apply(u, 1, sum)

nusvm[t] <- sqrt((mean((k - y)^2)))

corrv[t] <- cor(k, y)

t <- t + 1

}

#pick best model

rmses <- nusvm

mn <- which.min(rmses)

model <- out[[mn]]

#get and normalize coefficients

q <- t(model$coefs) %*% model$SV

q[which(q<0)]<-0

w <- (q/sum(q))

mix_rmse <- rmses[mn]

mix_r <- corrv[mn]

newList <- list("w" = w, "mix_rmse" = mix_rmse, "mix_r" = mix_r)

}

#' do permutations

#' @param perm Number of permutations

#' @param X cell-specific gene expression

#' @param y mixed expression per sample

#' @export

doPerm <- function(perm, X, Y){

itor <- 1

Ylist <- as.list(data.matrix(Y))

dist <- matrix()

while(itor <= perm){

#print(itor)

#random mixture

yr <- as.numeric(Ylist[sample(length(Ylist),dim(X)[1])])

#standardize mixture

yr <- (yr - mean(yr)) / sd(yr)

#run CIBERSORT core algorithm

result <- CoreAlg(X, yr)

mix_r <- result$mix_r

#store correlation

if(itor == 1) {dist <- mix_r}

else {dist <- rbind(dist, mix_r)}

itor <- itor + 1

}

newList <- list("dist" = dist)

}

#' Main functions

#' @param sig_matrix file path to gene expression from isolated cells

#' @param mixture_file heterogenous mixed expression

#' @param perm Number of permutations

#' @param QN Perform quantile normalization or not (TRUE/FALSE)

#' @export

CIBERSORT <- function(sig_matrix, mixture_file, perm=0, QN=TRUE){

library(e1071)

library(parallel)

library(preprocessCore)

#read in data

X <- read.table(sig_matrix,header=T,sep="\t",row.names=1,check.names=F)

Y <- read.table(mixture_file, header=T, sep="\t", row.names=1,check.names=F)

X <- data.matrix(X)

Y <- data.matrix(Y)

#order

X <- X[order(rownames(X)),]

Y <- Y[order(rownames(Y)),]

P <- perm #number of permutations

#anti-log if max < 50 in mixture file

if(max(Y) < 50) {Y <- 2^Y}

#quantile normalization of mixture file

if(QN == TRUE){

tmpc <- colnames(Y)

tmpr <- rownames(Y)

Y <- normalize.quantiles(Y)

colnames(Y) <- tmpc

rownames(Y) <- tmpr

}

#intersect genes

Xgns <- row.names(X)

Ygns <- row.names(Y)

YintX <- Ygns %in% Xgns

Y <- Y[YintX,]

XintY <- Xgns %in% row.names(Y)

X <- X[XintY,]

#standardize sig matrix

X <- (X - mean(X)) / sd(as.vector(X))

#empirical null distribution of correlation coefficients

if(P > 0) {nulldist <- sort(doPerm(P, X, Y)$dist)}

#print(nulldist)

header <- c('Mixture',colnames(X),"P-value","Correlation","RMSE")

#print(header)

output <- matrix()

itor <- 1

mixtures <- dim(Y)[2]

pval <- 9999

#iterate through mixtures

while(itor <= mixtures){

y <- Y[,itor]

#standardize mixture

y <- (y - mean(y)) / sd(y)

#run SVR core algorithm

result <- CoreAlg(X, y)

#get results

w <- result$w

mix_r <- result$mix_r

mix_rmse <- result$mix_rmse

#calculate p-value

if(P > 0) {pval <- 1 - (which.min(abs(nulldist - mix_r)) / length(nulldist))}

#print output

out <- c(colnames(Y)[itor],w,pval,mix_r,mix_rmse)

if(itor == 1) {output <- out}

else {output <- rbind(output, out)}

itor <- itor + 1

}

#save results

write.table(rbind(header,output), file="CIBERSORT-Results.txt", sep="\t", row.names=F, col.names=F, quote=F)

#return matrix object containing all results

obj <- rbind(header,output)

obj <- obj[,-1]

obj <- obj[-1,]

obj <- matrix(as.numeric(unlist(obj)),nrow=nrow(obj))

rownames(obj) <- colnames(Y)

colnames(obj) <- c(colnames(X),"P-value","Correlation","RMSE")

obj

}

#install.packages('e1071')

#if (!requireNamespace("BiocManager", quietly = TRUE))

# install.packages("BiocManager")

#BiocManager::install("preprocessCore")

#if (!requireNamespace("BiocManager", quietly = TRUE))

# install.packages("BiocManager")

#BiocManager::install("limma")

library("limma") #引用包

setwd("C:\\Users\\jindi1996\\Desktop\\BCaGenePair\\19.CIBERSORT") #设置工作目录

expFile="symbol.txt" #表达输入文件

#读取输入文件，并对输入文件整理

rt=read.table(expFile,sep="\t",header=T,check.names=F)

rt=as.matrix(rt)

rownames(rt)=rt[,1]

exp=rt[,2:ncol(rt)]

dimnames=list(rownames(exp),colnames(exp))

data=matrix(as.numeric(as.matrix(exp)),nrow=nrow(exp),dimnames=dimnames)

data=avereps(data)

#去除正常样品

if(grepl("-",colnames(data)[ncol(data)])){

group=sapply(strsplit(colnames(data),"\\-"),"[",4)

group=sapply(strsplit(group,""),"[",1)

group=gsub("2","1",group)

data=data[,group!=1]

}

#去除低表达基因

data=data[rowMeans(data)>0,]

#数据矫正

v <-voom(data, plot = F, save.plot = F)

out=v$E

out=rbind(ID=colnames(out),out)

write.table(out,file="uniq.symbol.txt",sep="\t",quote=F,col.names=F) #输出文件

#运行CIBERSORT，得到免疫细胞含量结果

source("immunePair19.CIBERSORT.R")

results=CIBERSORT("ref.txt", "uniq.symbol.txt", perm=100, QN=TRUE)

pFilter=0.05 #CIBERSORT结果过滤条件

setwd("C:\\Users\\jindi1996\\Desktop\\BCaGenePair\\19.CIBERSORT") #设置工作目录

rt=read.table("CIBERSORT-Results.txt",sep="\t",header=T,row.names=1,check.names=F) #读取文件

data=rt[rt[,"P-value"]<pFilter,]

data=data[,1:(ncol(rt)-3)]

rownames(data)=gsub("(.*?)\\-(.*?)\\-(.*?)\\-(.*?)\\-.*","\\1\\-\\2\\-\\3",rownames(data))

#读取risk文件

risk=read.table("tcgaRisk.txt",header=T,sep="\t",row.names=1,check.names=F)

sameSample=intersect(row.names(data),row.names(risk))

data=data[sameSample,]

risk=risk[sameSample,]

riskHigh=risk[risk$risk=="high",]

riskLow=risk[risk$risk=="low",]

dataHigh=data[row.names(riskHigh),]

dataLow=data[row.names(riskLow),]

newData=rbind(cbind(dataHigh,risk="High-risk"),cbind(dataLow,risk="Low-risk"))

#免疫细胞相关性分析，输出图形结果

risk="risk"

dotCol=c("red","blue")

outTab=data.frame()

for(cell in colnames(newData[,1:(ncol(newData)-1)])){

rt1=rbind(cell=newData[,cell],risk=newData[,risk])

rt1=as.matrix(t(rt1))

wilcoxTest=wilcox.test(cell ~ risk, data=rt1)

pValue=wilcoxTest$p.value

sig=ifelse(pValue<0.001,"***",ifelse(pValue<0.01,"**",ifelse(pValue<0.05,"*"," ")))

outTab=rbind(outTab,cbind(cell=cell,pValue=pValue,sig))

pval=0

if(pValue<0.001){

pval=signif(pValue,4)

pval=format(pval, scientific = TRUE)

}else{

pval=sprintf("%0.3f",pValue)

}

if(pValue<0.05){

b = boxplot(cell ~ risk, data = rt1,outline = FALSE, plot=F)

yMin=min(b$stats)

yMax = max(b$stats/5+b$stats)

ySeg = max(b$stats/10+b$stats)

ySeg2 = max(b$stats/12+b$stats)

n = ncol(b$stats)

pdf(file=paste0(cell,".pdf"),width=6,height=5)

par(mar = c(4,7,3,3))

boxplot(cell ~ risk, data = rt1, ylab = cell,col=dotCol,xlab="",names=c("High risk","Low risk"),

cex.main=1.2, cex.lab=1, cex.axis=1, ylim=c(yMin,yMax), outline = FALSE)

segments(1,ySeg, n,ySeg);segments(1,ySeg, 1,ySeg2);segments(n,ySeg, n,ySeg2)

text((1+n)/2,ySeg,labels=paste0("p=",pval),cex=1,pos=3)

dev.off()

}

}

write.table(outTab,file="corStat.txt",sep="\t",row.names=F,quote=F)

#输出雷达图需要的输入文件

fmsb=rbind(colMeans(dataHigh),colMeans(dataLow))

row.names(fmsb)=c("High","Low")

write.table(cbind(id=rownames(fmsb),fmsb),file="fmsbInput.txt",sep="\t",quote=F,row.names=F)

#install.packages("fmsb")

library(fmsb)

setwd("C:\\Users\\jindi1996\\Desktop\\BCaGenePair\\19.CIBERSORT") #设置工作目录

data=read.table("fmsbInput.txt",header=T,sep="\t",row.names=1,check.names=F) #读取输入文件

data=rbind(rep(max(data),22),rep(0,22),data)

#定义图形顺序

sortCellNames= c("B cells memory",

"B cells naive",

"Dendritic cells activated",

"Dendritic cells resting",

"Eosinophils",

"Macrophages M0",

"Macrophages M1",

"Macrophages M2",

"Mast cells activated",

"NK cells resting",

"Plasma cells",

"Mast cells resting",

"T cells CD4 memory activated",

"T cells CD4 naive",

"T cells CD4 memory resting",

"Monocytes",

"Neutrophils",

"NK cells activated",

"T cells CD8","T cells follicular helper",

"T cells gamma delta",

"T cells regulatory (Tregs)")

data=data[,sortCellNames]

#定义颜色

colors=c("red","blue")

#定义显著性

corStat=read.table("corStat.txt",header=T,sep="\t",row.names=1,check.names=F)

corStat=corStat[sortCellNames,]

colnames(data)=paste0(colnames(data),corStat$sig)

#输出结果

pdf(file="radar.pdf",height=8,width=8)

radarchart( data , axistype=1 ,

pcol=colors, #设置颜色

plwd=2 , #线条粗线

plty=1, #虚线，实线

cglcol="grey", #背景线条颜色

cglty=1, #背景线条虚线，实线

caxislabels=seq(0,1,0.05), #坐标刻度

cglwd=0.8, #背景线条粗细

axislabcol="grey", #刻度颜色

vlcex=0.75 #字体大小

)

legend("topright",legend=rownames(data[-c(1,2),]), bty = "n", pch=20 , col=colors ,cex=1.2, pt.cex=2)

dev.off()

#if (!requireNamespace("BiocManager", quietly = TRUE))

# install.packages("BiocManager")

#BiocManager::install("limma")

#if (!requireNamespace("BiocManager", quietly = TRUE))

# install.packages("BiocManager")

#BiocManager::install("org.Hs.eg.db")

library(limma)

library(org.Hs.eg.db)

setwd("C:\\Users\\jindi1996\\Desktop\\BCaGenePair\\22.preGSEA") #设置工作目录

expFile="symbol.txt" #表达文件名字

riskFile="tcgaRisk.txt" #风险文件名字

#读取文件

rt=read.table(expFile,sep="\t",header=T,check.names=F)

#如果一个基因占了多行，取均值

rt=as.matrix(rt)

rownames(rt)=rt[,1]

exp=rt[,2:ncol(rt)]

dimnames=list(rownames(exp),colnames(exp))

data=matrix(as.numeric(as.matrix(exp)),nrow=nrow(exp),dimnames=dimnames)

data=avereps(data)

##去除正常样品

if(grepl("-",colnames(data)[ncol(data)])){

group=sapply(strsplit(colnames(data),"\\-"),"[",4)

group=sapply(strsplit(group,""),"[",1)

group=gsub("2","1",group)

data=data[,group!=1]

colnames(data)=gsub("(.*?)\\-(.*?)\\-(.*?)\\-(.*?)\\-.*","\\1\\-\\2\\-\\3",colnames(data))

}

#读取risk文件

risk=read.table(riskFile,header=T,sep="\t",row.names=1,check.names=F)

sameSample=intersect(colnames(data),row.names(risk))

data=data[,sameSample]

risk=risk[sameSample,]

#low risk和high risk组比较

riskLow=risk[risk$risk=="low",]

riskHigh=risk[risk$risk=="high",]

dataLow=data[,row.names(riskLow)]

dataHigh=data[,row.names(riskHigh)]

meanLow=rowMeans(dataLow)

meanHigh=rowMeans(dataHigh)

meanLow[meanLow<0.00001]=0.00001

meanHigh[meanHigh<0.00001]=0.00001

logFC=log2(meanHigh/meanLow)

logFC=sort(logFC)

genes=names(logFC)

#输出GSEA需要的输入文件

entrezIDs <- mget(genes, org.Hs.egSYMBOL2EG, ifnotfound=NA) #找出基因对应的id

id=cbind(genes,entrezIDs,logFC)

write.table(file="id.txt",id,sep="\t",quote=F,row.names=F)

#if (!requireNamespace("BiocManager", quietly = TRUE))

# install.packages("BiocManager")

#BiocManager::install("fgsea")

#install.packages("ggplot2")

library(fgsea)

library(ggplot2)

library(data.table)

gmtFile="c2.cp.kegg.v7.0.symbols.gmt" #GSEA数据集文件

setwd("C:\\Users\\jindi1996\\Desktop\\BCaGenePair\\22.preGSEA") #设置工作目录

rt=read.table("id.txt",sep="\t",header=T,check.names=F) #读取输入文件

rt=rt[is.na(rt[,"entrezIDs"])==F,]

geneFC=rt$logFC

gene=rt$genes

names(geneFC)=gene

#读取gmt文件

gmt=gmtPathways(gmtFile)

#富集分析

fgseaRes=fgsea(pathways = gmt,

stats = geneFC,

minSize=15,

maxSize=500,

nperm=10000)

#对富集结果过滤，输出显著富集的结果

fgseaRes=fgseaRes[order(pval),]

fgseaRes=fgseaRes[fgseaRes$padj < 0.05,]

fwrite(fgseaRes, file="fgseaRes.txt", sep="\t")

#绘制GSEA富集图

for(i in 1:nrow(fgseaRes)){

term=as.character(fgseaRes[i,"pathway"])

gseaPlot=plotEnrichment(gmt[[term]],geneFC) + labs(title=term)

pdf(file=paste0(term,".pdf"),width=5.5,height=4.3)

print(gseaPlot)

dev.off()

}

#install.packages("ggplot2")

showTermNum=15 #展示前50个Term

library(ggplot2) #引用ggplot2这个包

setwd("C:\\Users\\Xiaonan Zheng\\Desktop\\paper\\Under Submission\\immune gene pair-MIBC\\BCaGenePair\\22.preGSEA") #设置工作目录

data = read.table("fgseaRes.txt",header=T,sep="\t") #读取输入文件

#如果富集的Term数目大于50，显示前50个;如果小于50，显示所有Term

if(nrow(data)>showTermNum){

data=data[1:showTermNum,]

}

#气泡图按照NES排序

data=data[order(data$NES,decreasing=T),]

data$pathway = factor(data$pathway,levels=rev(as.character(data[,"pathway"])))

#绘制气泡图

p = ggplot(data,aes(NES,pathway))

p = p + geom_point(aes(color=padj,size=size) )

pr = p +

scale_color_gradient(low="red",high="blue")+

labs(color="padj",size="size",x="Normalized enrichment score",y="Term")+

guides(color = guide_colorbar(order = 2), size = guide_legend(order = 1))+

theme_bw()

#保存图片

ggsave("bubble.pdf",width=9,height=6)
